# The Associations Between Neuropsychiatric Symptoms and Cognition in People with Dementia: A Systematic Review and Meta-Analysis

**DOI:** 10.1007/s11065-023-09608-0

**Published:** 2023-07-21

**Authors:** Julieta Sabates, Wei-Hsuan Chiu, Samantha Loi, Amit Lampit, Hanna M. Gavelin, Terence Chong, Nathalie Launder, Anita M. Y. Goh, Amy Brodtmann, Nicola Lautenschlager, Alex Bahar-Fuchs

**Affiliations:** 1 The University of Melbourne, 151 Barry Street, Carlton, VIC Australia; 2 Royal Melbourne Hospital, Parkville, Australia; 3 Department of Psychology, Umea University, Umea, Sweden; 4 St Vincent’s Hospital, Melbourne, Australia; 5 National Ageing Research Institute &, The University of Melbourne, Parkville, Australia; 6 Central Clinical School, Cognitive Health Initiative, Monash University, Melbourne, Australia; 7 Deakin University, Melbourne, Australia

**Keywords:** Dementia, Neuropsychiatric symptoms, Cognition

## Abstract

Most people with dementia experience neuropsychiatric symptoms (NPS), including anxiety, depression or disinhibition. There is growing interest in the relationship between NPS and cognitive impairment, but data is still limited. This study aimed to investigate the specific associations between NPS and cognition in people with dementia. MEDLINE, EMBASE and PsycINFO were searched for published, peer-reviewed studies of associations between at least one NPS and one cognitive ability in people with dementia. The quality of the studies was assessed with the NIH National Heart, Lung and Blood Institute’s quality assessment tools. A meta-analysis was conducted using Robumeta package for R. Ninety studies were included. We found significant associations between NPS, global cognition and cognitive domains, e.g. apathy was associated with global cognitive and memory impairment; dysphoria was associated with worse attention; delusions with executive dysfunction. Increased NPS in people with dementia are associated with worse cognitive performance. There were few studies looking at associations between some neuropsychiatric clusters and cognitive abilities, and there was little research on causal relationships. Our review was limited by the inclusion of studies that reported associations in specific formats, and most included people with a diagnosis of Alzheimer’s disease (AD). However, given the large number of studies, this is unlikely to have biased results. More research is needed that includes diverse people with different dementia syndromes. Registration: PROSPERO 2020 CRD42020165565.

**Supplementary Information:**

The online version contains supplementary material available at 10.1007/s11065-023-09608-0.

## Introduction

Dementia is a major cause of disability worldwide and in Australia, the leading cause of death in women (Australian Institute of Health & Welfare, 2022). Globally, an estimated 57.4 million people were living with dementia in 2019, and this number is expected to increase to 152.8 million by 2050 (Nichols et al., 2022).

Neuropsychiatric symptoms (NPS) are behavioural and psychological disturbances highly prevalent in people with dementia (Prince et al., 2014), with around 97% of people with dementia reported experiencing at least one NPS since the onset of their dementia syndrome (Steinberg et al., 2008). Examples of NPS are apathy, irritability, aggression (verbal or physical), anxiety, depression, psychosis, sleep disturbances, eating disturbances, aberrant motor behaviour, wandering and disinhibition. NPS have been associated with worse outcomes (Rabins et al., 2013), lower quality of life (Appelhof et al., 2017) and greater caregiver distress (Millenaar et al., 2016), which is known to lead to higher rates of hospitalisation (de Vugt et al., 2005) and unwanted relocation to long term care (Gaugler et al., 2009).

Because of the adverse effects often associated with pharmacological treatment, current clinical guidelines recommend non-pharmacological interventions as the first-line approach for managing NPS (Livingston et al., 2020). However, no specific non-pharmacological treatment approach has been found to reduce the frequency or severity of these symptoms significantly and consistently. There are multiple theories behind the development of NPS including insufficient understanding of the cognitive and other mechanisms that contribute to NPS. Improving our understanding of the links between specific cognitive processes and specific NPS is important in guiding the development of effective non-pharmacological treatment options.

A considerable amount of literature has been published on the associations between NPS and cognition in people with dementia. However, much of the evidence has come from cross-sectional and longitudinal studies, and to our knowledge, to date, there have been no systematic reviews that have synthesised the evidence. This review addresses this gap, and our overarching goal was to investigate and characterise the associations between neuropsychiatric symptoms (as a group and individually) with overall cognitive function and with specific cognitive abilities in people with dementia, and to explore potential moderators of these relationships (e.g. dementia syndrome, age of symptom onset, severity of dementia).

We aimed to:

1. Identify whether neuropsychiatric symptoms (e.g. apathy, depression, agitation) are linked with cognitive functioning in people with dementia.
2. Examine whether such associations differ across symptoms and cognitive domains.
3. Compare these associations across key dementia syndromes and patient groups.
4. Identify other potential moderators of such associations.

## Methods

This systematic review was prospectively registered on the Prospective Register of Systematic Reviews (CRD42020165565). The Preferred Reporting Items for Systematic Reviews and Meta-Analysis 2020 statement provided the framework for reporting.

### Eligibility Criteria and Information Sources

MEDLINE, EMBASE and PsycINFO were searched from inception to 14 December 2021 for potentially eligible peer-reviewed papers describing case-series, case–control studies (cases need to be people with dementia with any of the specific NPS) cross-sectional studies, cohort studies and intervention studies that investigated associations between cognition and NPS in people with dementia of any aetiology.

### Search Strategy

Search terms were related to dementia syndromes, neuropsychiatric symptoms and cognitive domains. Some of the search terms were as follows: Dement*, Alzheim*, AD, Frontotemporal, FTD, Neurocognitive disorder*, Parkinson*, Huntington*, Lewy Bod*, BPSD, behavior*, psychology*, apath*, depress*, anxi*, aggress*, agitat*, aberrant motor, irritab*, disinhibit*, eat* disturbance*, eat* problem*, sleep* disturbance*, psychotic, psychosis, hallucinat*, delu*, wander*, impuls*, mood, neuropsychology*, cognition, cognitive function*, cognitive skill*, cognitive ability*, cognitive impairment*, cognitive dysfunction*, cognitive problem*, cognitive profile, cognitive deficit*, memory, attention*, executive, speed, language, visuospatial, visuoconstruction*, fluency, learning, associate*, relat* and correlate*.

See the full search strategy (S1) published as supplementary material online attached to the electronic version of this paper.

### Selection Process

Studies were independently screened and selected for inclusion using a single Covidence library by two reviewers, with a third reviewer resolving discrepancies.

Studies were eligible if they provided at least one measure of association for the relationship between cognition and neuropsychiatric symptoms. Measures of association could be reported either as correlation coefficients or as mean differences on continuous measures. Authors of studies that did not provide these data were contacted via email. If data were unavailable, the study was excluded from the review.

Only studies conducted in humans were included. Studies published in English or studies for which we were able to request and obtain a manuscript in English were included.

### Data Collection Process and Data Items

After the selection of included studies, demographic information was extracted. For intervention or longitudinal studies, only baseline data were extracted since studying longitudinal relationships, which attempts to address issues of a temporal nature (e.g. cause and effect), was beyond the scope of the current review.

Subgroup analysis based on dementia syndrome or diagnosis was conducted.

### Outcomes

The main outcome of interest in this review was the association between global cognition or specific cognitive abilities (as measured by cognitive test performance) and overall and specific neuropsychiatric symptoms.

Given the heterogeneous nature of NPS and the lack of a universally accepted classification of these, for the purposes of this review, NPS were grouped in six clinically driven clusters of symptoms and behaviours:

1. Affect (depression, dysphoria, anxiety, elation/euphoria, apathy, indifference)
2. Aggression (e.g. agitation, aggression, irritability, lability)
3. Circadian rhythms (e.g. night-time behaviours, appetite/eating disturbances)
4. Executive dysfunction (e.g. disinhibition, social inappropriateness)
5. Motor disturbances (e.g. wandering, repetitive movements)
6. Psychosis (e.g. delusions, hallucinations)

For the purposes of this review, the following cognitive domains were considered: (1) attention, (2) executive functions, (3) memory, (4) working memory, (5) semantic knowledge, (6) social cognition, (7) speed of information processing, (8) visuospatial skills.

### Study Risk of Bias Assessment

Methodological quality of included studies was assessed with the NIH National Heart, Lung and Blood Institute’s Quality Assessment Tool for Observational Cohort and Cross-Sectional Studies, Quality Assessment of Case–Control Studies and Quality Assessment Tool for Case Series Studies (National Institutes of Health, 2014a, b).

These assessments were conducted by two reviewers, with a third reviewer resolving discrepancies.

### Synthesis Methods

Outcome data from primary reports were extracted and converted to standardised mean difference (SMD, calculated as Hedges’ g) with 95% confidence interval (CI) of difference in cognitive performance between the exposure groups or correlation coefficient. R package *Robumeta* was used to synthesise the results.

Pooling of data was conducted for overall as well as symptom- and domain-specific measures. When a study reported both mean differences and correlations for the same measures and population, the correlation scores were used (to avoid double-counting participants).

When a measure was used to evaluate several domains (e.g. NPI Total or Frontal Behavioural Inventory Total score), it was classified under ‘Overall neuropsychiatric symptoms’. If a measure assessed dysphoria and apathy, for example, it was classified under ‘affective’ and used in the meta-analysis of broad domains, but not in the meta-analysis of narrow domains (e.g. dysphoria or apathy).

For the purposes of this review, a negative association was obtained when higher scores on measures of NPS were associated with lower scores on measures of cognitive performance.

### Reporting Bias Assessment

R package *Publication Bias* was used, which allows for visual inspection of funnel plots and testing with an analogue of Egger’s test.

## Results

### Study Selection

**Fig. 1:**
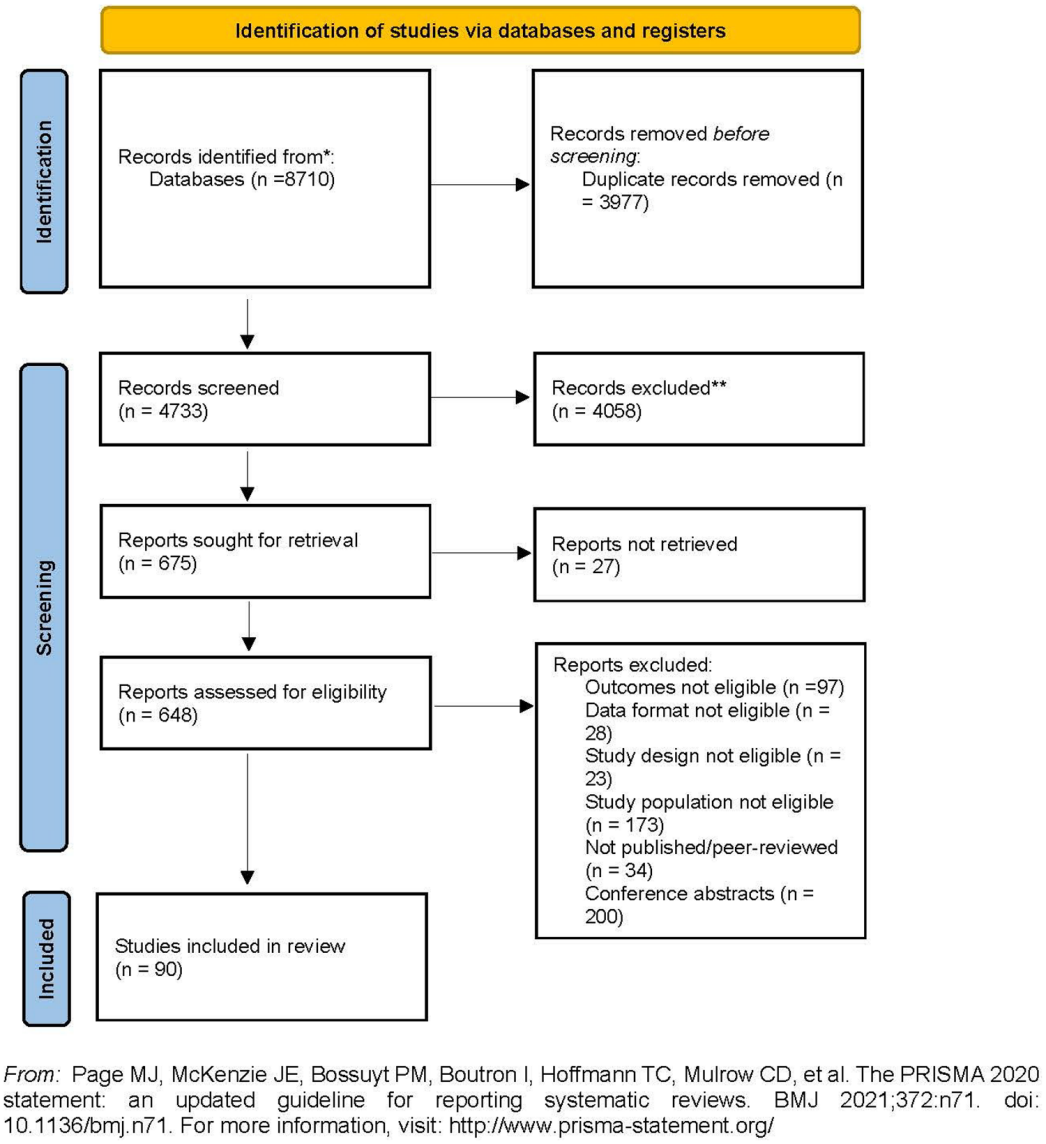
Study selection flow chart

Initial search yielded 8710 items, and after removing duplicates, 4733 records were screened for inclusion. Six hundred and forty-eight studies were assessed for eligibility in the full-text review. Ninety studies met the criteria and were included. Figure 1 presents a flow chart showing the study selection process.

### Study Characteristics

Ninety studies were included. Of these studies, 64 (71.1%) were cross-sectional design, 16 (17.8%) were longitudinal and 10 (11.1%) were case–control studies. No case-series studies were eligible for this review.

Included studies were published between 1991 and 2021. They were conducted in 23 countries, with the largest number of studies (*k* = 26) conducted in the USA, followed by Brazil and Italy (*k* = 8 each).

The total number of participants with dementia included in the studies was 26,893, with sample sizes ranging from 13 to 6265. Participants in all studies had a diagnosis of dementia. In most studies (*k* = 57, 63.3%), only one suspected clinical dementia syndrome (AD) was included. In 18 studies (20%), participants with dementia were suspected to have AD or another type of dementia (e.g. vascular dementia, frontotemporal dementia or dementia with Lewy bodies (DLB), among other types). There were four studies (4.4%) focusing on people with Parkinson’s disease dementia (PDD), and two (2.2%) on people with DLB. Three studies (3.3%) included people with either of these two types of dementia. Two studies (2.2%) included only people with post-stroke or vascular dementia. One (1.1%) study focused on people with primary progressive aphasia. Three studies did not report dementia syndrome in their included participants.

In 64.4% of the studies (*k* = 58), at least 51% of participants with dementia were reported as female. The mean age of participants ranged between 58 and 86.3 years old. Most (84.4%) of the studies did not report whether the sample included people with younger-onset or older-onset dementia, that is, the age of onset of the dementia was not reported. They also did not specify whether participants were in residential care or living in the community, or whether they were taking any medications. Participants’ ethnicity was also infrequently reported, with only 22 (24.4%) studies reporting participants’ nationality, race or cultural background.

Sixty-eight studies (75.6%) reported the Mini-Mental State Examination (MMSE) (Folstein et al., 1983) mean scores in participants with dementia, (range 7.8–26.1). Thirty-seven (41.1%) studies used the Neuropsychiatric Inventory (NPI) to measure at least one neuropsychiatric symptom.

A table summarising the characteristics of included studies (Table 1) is presented below. See the full table (S2) published as supplementary material online attached to the electronic version of this paper.

**Table 1:** Characteristics of included studies

**Study ID**	**Country**	**Sample size (dementia)**	**Gender ratio (dementia; % female)**	**Mean age (dementia)**	**Young vs late onset/age of onset**	**Dementia syndrome**	**Severity (CDR)/global cognition (MMSE)**
Akyol et al. (2020)	Turkey	106	46.23%	72.25	NR	AD, VaD, FTD	MMSE = 15.77
Balci et al. (2011)	Turkey	26	80.80%	76.23	NR	AD	MMSE = 15.69
Bhat et al. (2021)	India	76	28.90%	58.05	NR	VaD	CDR = 1.65
Benedict et al. (1999)	USA	13	92.00%	73.43	NR	VaD	MMSE = 22.4
Breitve et al. (2018)	Norway	196	37.00%	76.06	NR	AD, DLB	CDR = 0.87
Bronnick et al. (2011)	Norway	172	38.00%	73.21	NR	PDD	MMSE = 20.11
Bylsma et al. (1994)	USA	180	58.00%	72.38	Late (68.35)	AD	CDR = 1.2
Camargo et al. (2018)	Brazil	40	35.00%	70.08	NR	PDD	SCOPA-Cog = 11.25
Chwiszczuk et al. (2017)	Norway	246	56.56%	75.52	NR	AD, DLB, PDD	MMSE = 23.64
Contador-Castillo et al. (2009)	Spain	23	65.22%	75.31	NR	AD	MMSE = 22.73
D’Antonio et al. (2019)	Italy	32	50.00%	74.28	NR	AD	MMSE = 19.63
de Oliveira et al. (2015a) (‘Correlations’)	Brazil	217	67.70%	78	Late (73.19)	AD	CDR 1, 2, 3
de Oliveira et al. (2015b)2 ‘contrasts’	Brazil	39	56.41%	78	Late	AD, PDD, LBD	MMSE = 17.52
de Oliveira et al. (2020)	Brazil	51	39.20%	77.76	NR	PD, LBD	MMSE = 15.82, CDR = 11.46
DeMichele-Sweet et al. (2011)	USA	2317	57.00%	78.4	Late (72.3)	AD	MMSE 17.7
de Paula et al. (2016)	Brazil	93	NR	74.57	NR	AD	MMSE = 20.59
Drijgers et al. (2011)	The Netherlands	260	56.00%	74.4	NR	AD	MMSE 20.3
Eikelboom et al. (2021)	The Netherlands	1090	52.40%	65.9	NR	AD	MMSE = 20.3
Eustace et al. (2001)	Ireland	150	69.00%	76.48	NR	AD	MMSE = 19.31
Fahlander et al. (1999)	Sweden	54	76.00%	83.93	NR	AD	MMSE 19.57
Fernández et al. (2010)	Spain	1014	65.00%	77.19	NR	AD	NR
Fernandez-Martinez et al. (2010)	Spain	99	56.60%	75.31	NR	AD	MMSE = 21.93
Fillit et al. (2021)	USA	6265	69.00%	80.47	NR	AD and other aetiologies	NR
Fitz and Teri (1994)	USA	91	55.00%	73.49	NR	AD	MDRS = 102.52
Flynn et al. (1991)	USA	33	9.00%	73.36	NR	AD / MID	MMSE = 16.5
Gallassi et al. (2001)	Italy	33	29.00%	77.1	NR	AD	MMSE = 15.4
Gallo et al. (2008)	USA	48	65.00%	79	NR	AD, VaD	MMSE = 23
Galynker et al. (1995)	USA	26	57.70%	78.8	NR	AD/multi-infarct dementia	MMSE = 16.8
Gilley et al. (1991)	USA	230	67.00%	71.54	NR	AD	MMSE 12.57
Grossi et al. (2013)	Italy	61	46.00%	69.54	NR	AD, PDD	MMSE = 22.13
Hallikainen et al. (2012)	Finland	236	51.30%	75.11	NR	AD	MMSE = 21.5
Harwood et al. (2000)	USA	114	63.00%	78.8	NR	AD	MMSE = 17.8
Hopkins and Libon (2005)	USA	48	NR	79.06	NR	VaD	MMSE = 21/33
Ito et al. (2007)	Japan	40	68.00%	77.8	NR	AD	CASI = 25.15
Janzing et al. (2005)	The Netherlands	60	88.30%	86.3	NR	NR	NR
Keator et al. (2019)	USA	58	50.00%	69.19	NR	PPA	NR
Kuzis et al. (1999)	Argentina	184	NR	70.88	NR	AD	MMSE = 22.49
Kwak et al. (2013)	Korea	230	61.73%	74.66	Late (71.88)	AD	CDR = 1.06
Lam et al. (2006)	China	125	58.40%	82.04	NR	AD, VaD	MMSE = 8.62
Lee et al. (2007)	Japan	50	86.00%	81.36	NR	AD	MMSE = 15.58
Lee et al. (2012)	Taiwan	127	40.15%	77	NR	PDD	MMSE = 17
Lee et al. (2019)	Korea	1247	56.60%	72.65	NR	AD	MMSE = 20.21
Levy et al. (1998)	USA	28	46.43%	63	NR	AD, FTD	MMSE = 16.5
Logsdon et al. (1998)	USA	193	49.00%	76.8	NR	AD	MMSE = 17.4
Lopez et al. (1991)	USA	17	76.00%	68	NR	AD	MMS = 18.7
Machado et al. (2020)	Brazil	32	59.00%	75.84	AOO = 71.14	DLB	MMSE = 17.72
Mariano et al. (2020)	Brazil	42	45.23%	68.31	YOD (64.08)	AD, FTD	MMSE = 25.06
McPherson et al. (2002)	USA	44	69.00%	75.72	NR	AD	MMSE = 22.57
Migliorelli et al. (1995)	Argentina	103	74.00%	73.58	Late (69.21)	AD	NR
Mizrahi et al. (2006)	Argentina	771	NR	71.1	NR	AD	MMSE = 21.8
Montagnese et al. (2022)	UK	284	49.00%	75.42	NR	DLB, PDD	MMSE = 26.05
Na et al. (2018)	South Korea	289	71.00%	77.68	NR	AD	CDR = 1, MMSE = 20.83
Naarding et al. (2007)	The Netherlands	54	46.00%	73.7	NR	Post-stroke dementia	MMSE = 19.8
Nagata et al. (2010)	Japan	50	78.00%	78.16	NR	AD	MMSE = 19.38
Nagata et al. (2017)	Japan	421	56.00%	77.9	NR	AD	MMSE = 15
Nakaaki et al. (2007)	Japan	42	52.38%	71.4	NR	AD	MMSE = 19.55
Nakaaki et al. (2008)	Japan	88	55.00%	71.23	NR	AD	MMSE = 19.76
Nakatsuka et al. (2014)	Japan	142	66.90%	80	NR	AD	MMSE 14.4
Onyike et al. (2007)	USA	316	NR	NR	NR	NR	MMSE = 14.32
Pagonabarraga et al. (2008)	Spain	30	43.00%	77.17	NR	PDD	MDRS = 102.33
Park et al. (2019)	Korea	1128	62.00%	73	NR	AD	MMSE = 20.1
Perneczky et al. (2009)	Germany	21	48.00%	71.1	Young (63.1)	DLB	MMSE = 20.8
Perri et al. (2014)	Italy	86	39.53%	69.06	NR	AD, frontal variant FTD, SIVD, LBD	CDR = 0.72, MMSE = 22.53
Perri et al. (2018)	Italy	20	55.00%	76.83	NR	AD	MMSE = 22.2
Pezzoli et al. (2019)	Italy	52	46.00%	70.98	NR	DLB	MMSE = 25.42
Qian et al. (2018)	Canada	900	44.00%	77.11	Late	AD	MMSE = 13.17
Quaranta et al. (2015)	Italy	108	64.00%	73.3	NR	AD	MMSE = 17.2
Reed et al. (1993)	USA	57	NR	74.43	NR	AD	MMSE = 19.4
Rochat et al. (2013)	Switzerland	30	NR	72.03	NR	AD	NR
Rolland et al. (2007)	France	682	72.00%	77.4	Late (74.1)	AD	MMSE = 20.1
Ross et al. (1998)	USA	1486	69.00%	77.01	NR	AD	MMSE = 16.62
Rozum et al. (2019)	USA	56	67.90%	85.68	NR	VaD	SCIP total = 154.77
Ruiz et al. (2018)	Spain	90	59.00%	76	NR	AD	MMSE = 22.4
Sánchez-Rodríguez (2004)	Spain	58	57.00%	73.34	Late	AD	MMSE = 24.3
Senanarong et al. (2005)	Thailand	73	72.60%	70.28	NR	AD	MMSE = 18.42
Serra et al. (2010)	Italy	54	66.67%	73.1	NR	AD	MMSE = 20.72
Shin et al. (2014)	Korea	63	73.00%	74.83	NR	AD	MMSE = 16.73
Soleman Hernandez et al. (2012)	Brazil	37	78.00%	78.8	NR	AD	MMSE = 17.2
Starkstein et al. (2005)	Argentina	150	90.00%	70.49	NR	AD	MMSE = 23.09
Starr and Lonie (2007)	UK	556	69.96%	77.3	NR	AD	MMSE = 19.2
Strauss and Sperry (2002)	USA	100	50.00%	75	NR	AD	MMSE = 18.55; CDR = 1.62
Sultzer et al. (1992)	USA	61	NR	73	NR	AD	MMSE = 10
Sultzer et al. (2014)	USA	88	19.00%	78	NR	AD	MMSE = 19.3
Van der Mussele et al. (2013)	Belgium	402	67.00%	80.1	Late (76.8)	AD	MMSE = 15.2, Global Deterioration Scale = 5.1
Van der Mussele et al. (2015)	Belgium	393	67.00%	80.1	Late (76.9)	AD	MMSE = 15.1
Wagner et al. (1995)	USA	614	69.00%	79	NR	Primarily AD	MMSE = 7.8
Welsh et al. (1996)	UK	18	94.40%	NR	NR	AD	NR
Wu (2014)	Taiwan	179	39.00%	78.4	NR	NR	NR
Yeager and Hyer (2008)	USA	68	60.00%	77	NR	AD, dementia-NOS	NR
Zahodne et al. (2015)	USA	517	57.00%	74.19	NR	AD	NR

*AD* Alzheimer’s disease, *BPSD* behavioural and psychological symptoms of dementia, *CDR* clinical dementia rating, *Dementia-NOS* dementia not otherwise specified, *DLB* dementia with Lewy bodies, *FTD* frontotemporal dementia, *MCI* mild cognitive impairment, *MMSE* Mini-Mental State Examination, *MID* multi-infarct dementia, *NPS* neuropsychiatric symptoms, *NR* not reported, *PPA* primary progressive aphasia, *PDD* Parkinson’s disease dementia, *SCOPA-Cog* Scales for Outcomes in PArkinson’s disease-COGnition, *SIVD* subcortical ischemic vascular dementia, *UK* United Kingdom, *USA* United States of America, *VaD* vascular dementia

### Study Quality

Different quality assessment forms were used for cross-sectional/longitudinal studies and for case–control studies. Eight items were selected from the Quality Assessment tool for cross-sectional and longitudinal studies, and nine from the quality assessment tool for case–control studies as being of relevance for this review. The number of ‘Yes’ answers was counted for each study and divided by the total number of relevant items. Studies that had up to 24.9% of positive answers were rated as ‘very low quality’, studies that had between 25 and 49.9% of positive answers were considered ‘low quality’, studies that had between 50% and 74.9% ‘Yes’ answers were considered ‘moderate quality’, and studies that had at least 75% positive answers were considered ‘high quality’.

According to this quality assessment, 60% of the included studies (*k* = 54) were considered high quality, 33.3% of studies (*k* = 30) were considered of moderate quality and 6.7% of the included studies (*k* = 6) were classified as low quality. No studies were considered very low quality.

Reviewers conducting the quality assessment assessed that most of the studies (96.7%; *k* = 87) clearly stated the research question or objective in the published manuscript; fewer studies (70%; *k* = 63) clearly specified and defined the study population. Around half of the studies (51.1%, *k* = 46) measured and adjusted statistically for key potential confounding variables.

Sixty-two out of 80 cross-sectional or longitudinal studies (77.5%) confirmed that all the participants had been selected or recruited from the same of similar populations. In some cases, this was not possible to be determined since authors did not report the time period during which they recruited participants. In these cases, studies received a NR (Not reported) in this item. Among the case–control studies, only 2 out of 10 studies received a Yes in this question (20%). Of the included 90 studies, outcome assessors were reported to have been blinded in only 12.2% (*k* = 11) of the cases.

See the full quality assessment tables (S3) published as supplementary material online attached to the electronic version of this paper.

### Results of Syntheses

Significant associations between NPS and cognition including subgroup analysis based on dementia syndrome are reported below. Results are presented in Hedge’s g. See a table with all results (S4) published as supplementary material online attached to the electronic version of this paper.

#### Overall NPS

The association between overall NPS and worse global cognition reached statistical significance when the whole sample was analysed (*g* =  − 0.36, 95% CI − 0.51 to − 0.22; *k* = 22) and remained significant when only participants with AD were included (*g* =  − 0.28, 95% CI − 0.44 to − 0.13; *k* = 14), but not when only a subsample with PD or DLB were analysed.

The analysis also revealed associations between overall NPS and cognitive domains. Specifically, greater/more NPS were shown to be associated with worse attention (*g* =  − 0.18, 95% CI − 0.25 to − 0.12; *k* = 6), worse executive function (*g* =  − 0.31, 95% CI − 0.45 to − 0.17; *k* = 12), worse memory (*g* =  − 0.21, 95% CI − 0.32 to − 0.10; *k* = 5) and worse semantic knowledge (*g* =  − 0.19, 95% CI − 0.33 to − 0.04; *k* = 8). The associations with global cognition, attention, executive function and memory remained significant when only the subsample of participants with AD was analysed. The same was true for the association between overall NPS and visuospatial skills but, as only two studies contributed data, the results may be unreliable (*g* =  − 0.14, 95% CI − 0.15 to − 0.12; *k* = 2).

Several significant associations were found between NPS clusters and symptoms and cognition.

#### Affect Cluster (Depression, Dysphoria, Anxiety, Elation/Euphoria, Apathy, Indifference)

A weak association was found between the affect cluster and worse global cognition (*g* =  − 0.09, 95% CI − 0.16 to − 0.01; *k* = 46), attention (*g* =  − 0.15, 95% CI − 0.26 to − 0.04; *k* = 14) and semantic knowledge (*g* =  − 0.15, 95% CI − 0.25 to − 0.05; *k* = 20). The association with attention was maintained when only a subsample of participants with AD was included in the analysis. However, results were not significant when only participants with DLB or with PPD were included.

Associations between the affect cluster and executive function, memory, social cognition, speed, visuospatial skills and working memory were not significant (*p* > 0.5) in the whole sample or in the analysed subsamples.

Within the affect cluster, the following significant associations were found:

##### Apathy

Apathy was associated with worse global cognition (*g* =  − 0.28, 95% CI − 0.41 to − 0.16; *k* = 24), as well as memory, semantic knowledge, visuospatial skills and working memory (*g* =  − 0.28 to − 0.16). The association between apathy and global cognition was maintained when only participants with AD were analysed. No significant links were found between apathy and any cognitive domains when only a subsample with PD or DLB was analysed.

##### Dysphoria

An association was found between dysphoria and worse attention (*g* =  − 0.10, 95% CI − 0.11 to − 0.09; *k* = 2). However, only two studies contributed data and therefore results may be unreliable.

#### Aggression Cluster (Agitation, Aggression, Irritability, Lability)

Elevated symptoms of aggression were associated with lower global cognition (*g* =  − 0.21, 95% CI − 0.33 to − 0.1; *k* = 16).

##### Agitation

Agitation was shown to be associated with worse global cognition (*g* =  − 0.24, 95% CI − 0.43 to − 0.04; *k* = 8), and this remained true when only participants with AD were analysed.

No significant associations were found between irritability, aggression or lability and any cognitive domains.

#### Circadian Rhythms Cluster (Night-Time Behaviours, Appetite/Eating Disturbances)

No associations were found between the circadian rhythms cluster and any cognitive domains.

Within this cluster, a significant association was found only between eating disturbances and worse visuospatial skills (*g* =  − 0.04, 95% CI − 0.07 to 0; *k* = 2) in the whole sample. However, only two studies contributed data, therefore results may be unreliable.

#### Executive Dysfunction Cluster (Disinhibition, Social Inappropriateness)

There were no statistically significant links between the d executive dysfunction cluster or the symptoms within this cluster (disinhibition and social inappropriateness) and any cognitive domains.

#### Motor Disturbances Cluster (Wandering, Repetitive Movements)

The motor disturbance cluster was associated with impaired global cognition (*g* =  − 0.46, 95% CI − 0.74 to − 0.18; *k* = 13). This was maintained when only participants with AD were analysed.

An association was also found between this cluster and worse working memory when only participants with AD were included (*g* =  − 0.48, 95% CI − 0.42 to − 0.11; *k* = 2). However, as only two studies contributed data, results may be unreliable.

No significant links were found between wandering or repetitive movements and cognitive domains.

#### Psychosis Cluster (Delusions, Hallucinations)

The psychosis cluster showed links with impairment in global cognition (*g* =  − 0.40, 95% CI − 0.54 to − 0.27; *k* = 36), executive function (*g* =  − 0.17, 95% CI − 0.33 to − 0.01; *k* = 16), and working memory (*g* =  − 0.22, 95% CI − 0.38 to − 0.05; *k* = 10).

The association between the psychosis cluster and global cognition was maintained when only participants with AD were included.

##### Delusions

Delusions were associated with worse global cognition (*g* =  − 0.31, 95% CI − 0.44 to − 0.18; *k* = 22), as well as with worse executive function, and semantic knowledge (*g* =  − 0.32 to − 0.24), and the association with global cognition was maintained when only a subsample of participants with AD was analysed.

##### Hallucinations

With regard to hallucinations specifically, an association between them and worse global cognition was found (*g* =  − 0.44, 95% CI − 0.69 to − 0.19; *k* = 20). When only a subsample with AD was analysed, associations between hallucinations and worse global cognition (*g* =  − 0.44) and working memory (*g* =  − 0.3) were found.

**Fig. 2:**
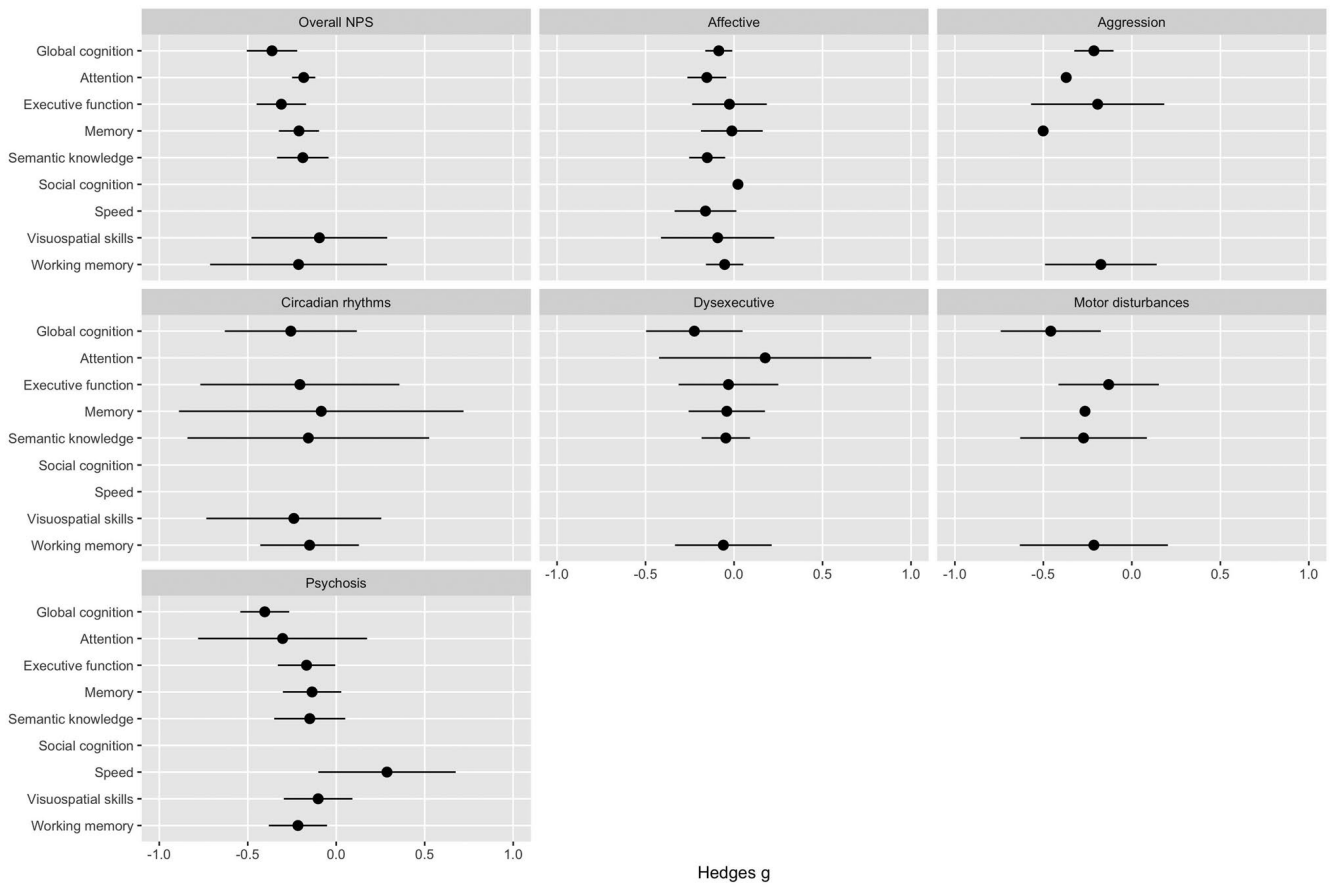
Forest plot NPS clusters and cognitive domains—all significant associations were negative (i.e. NPS were associated with worse cognitive performance)

**Fig. 3:**
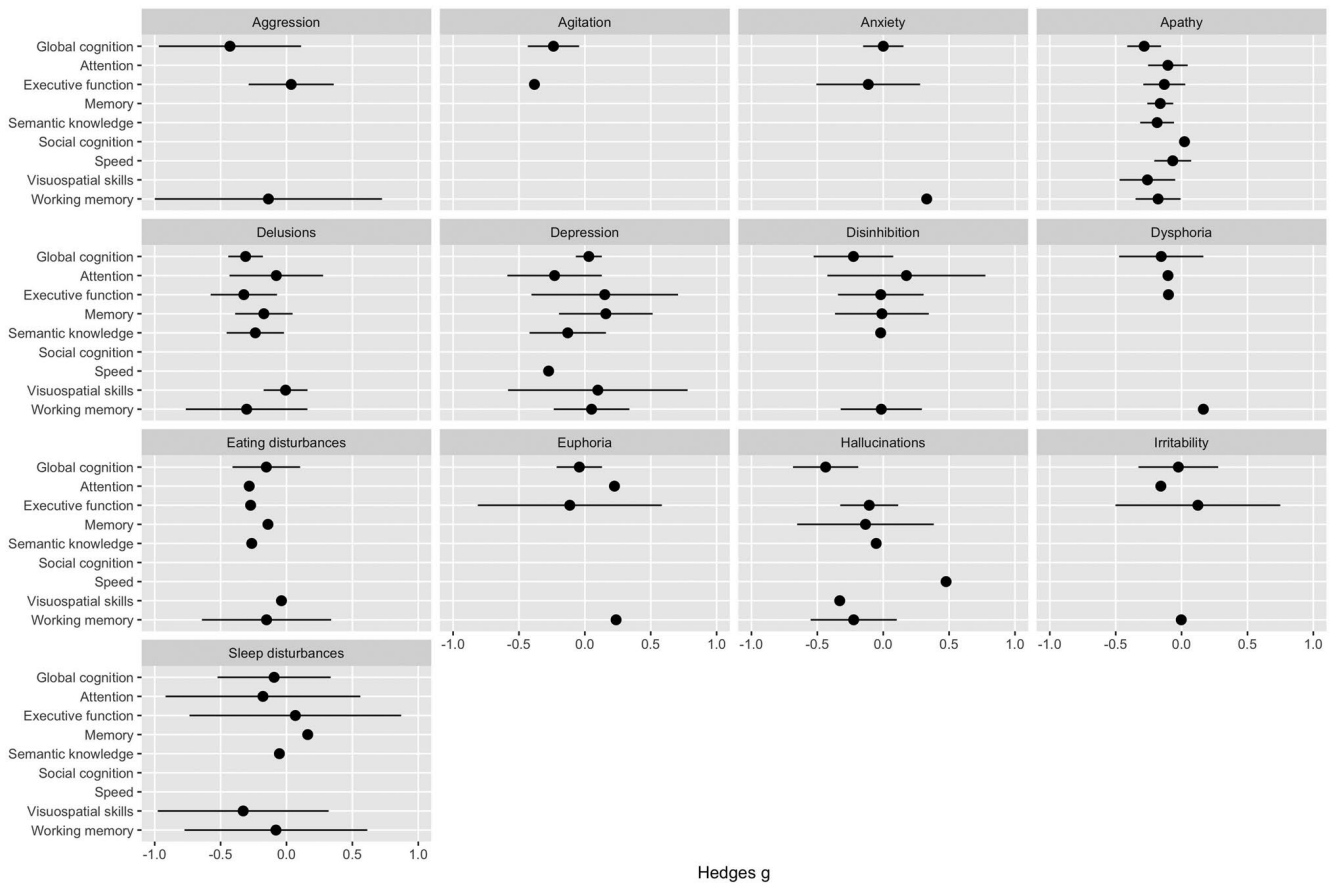
Forest plots specific NPS and cognitive domains—all significant associations were negative (i.e. NPS were associated with worse cognitive performance

Figure 2 summarises the associations between NPS clusters and cognitive domains. Figure 3 presents the associations between specific NPS and cognitive domains.

The CI for the following associations were broader than − 1 or 1 and are therefore not showing in the figure: attention and aggression, memory and aggression, memory and motor disturbances.

The CI for the following associations were broader than − 1 or 1 and are therefore not showing in the figure: attention and eating disturbances, semantic knowledge and eating disturbances, working memory and dysphoria, working memory and euphoria, attention and irritability.

### Reporting Biases

**Fig. 4:**
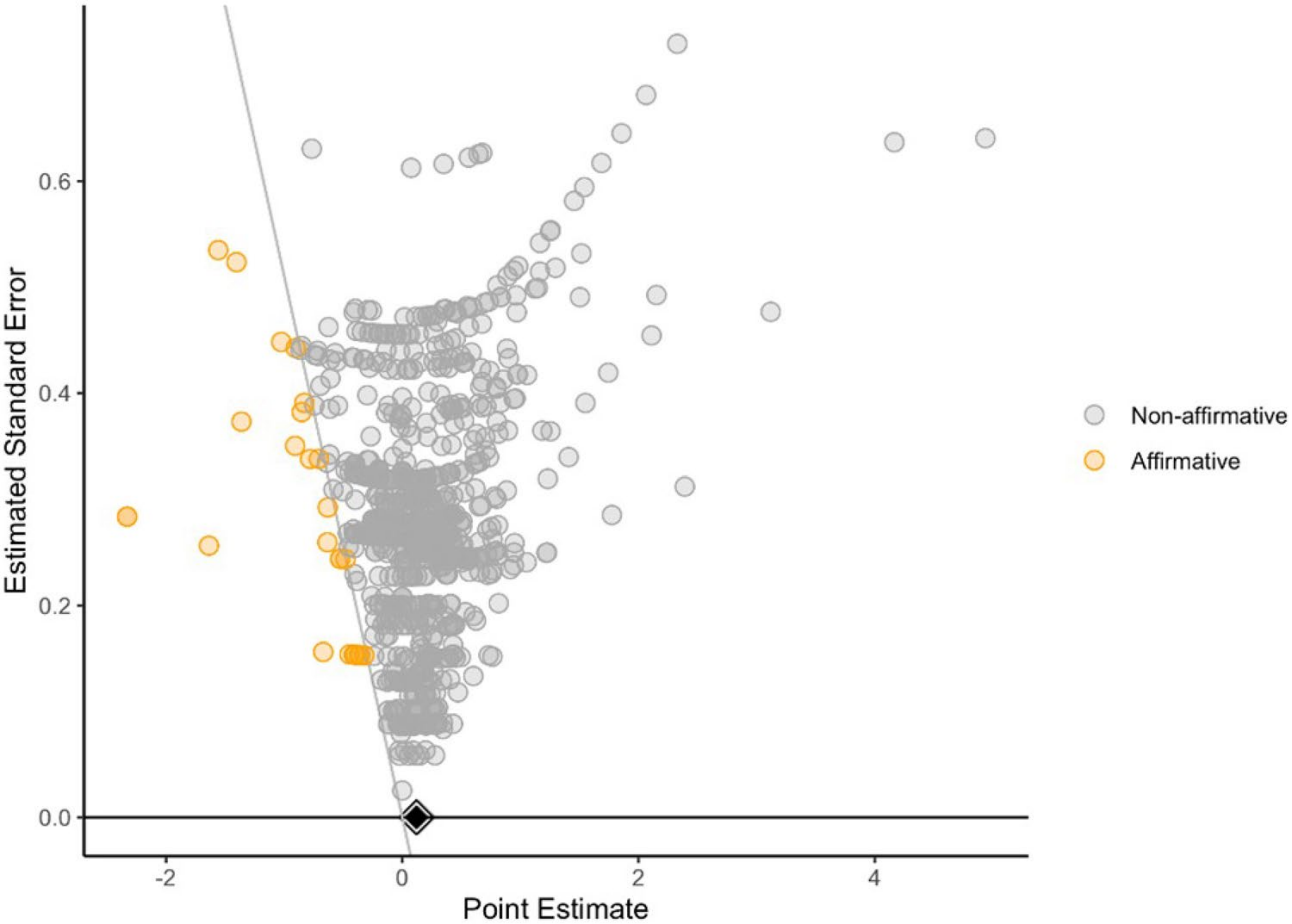
Funnel plot—no evidence of publication bias

In line with our hypothesis that there was a negative association between the analysed NPS and cognitive domains, we conducted a sensitivity analysis for publication bias favouring negative estimates in our meta-analysis using the ‘Publication Bias’ package in R. The funnel plot (Fig. 4) showed all point estimates from included studies and identified those which were systematically smaller than the entire set of point estimates (i.e. strongly favouring the hypothesised negative direction) and were identified as ‘affirmative’. With visual inspection of the funnel plot, the point estimates from included studies were equally spread across both positive and negative sides of the plot and most point estimates showed a small estimated standard error, suggesting that the meta-analysis may not be affected by publication bias. The funnel plot shows point that fall along distinct curves due to the inclusion of multiple effects based on the same study and, thus, the same sample.

Following visual inspection of the plot, we conducted a quantitative sensitivity analysis to examine if the affirmative point estimates were affecting the pooled point estimate indicated as a black diamond. The results suggested that the affirmative point estimates as a small part of the meta-analysis (*n* = 30) were unlikely to shift the pooled point estimate or its confidence intervals derived from a much larger sample size (*n* = 966), and therefore no further modification, such as removal of outliers, was needed. Both visualisation of funnel plot and severity test of publication bias suggested no clear evidence of publication bias in the current meta-analysis.

## Discussion

Over the last 30 years, several studies have investigated whether NPS are associated with impairment in cognition in people with dementia. This review examined those relationships by pooling together results from 90 published studies, and this is to the best of our knowledge the first study to systematically review these associations. See the full list of references of the included studies (S5) published as supplementary material online attached to the electronic version of this paper.

Our meta-analysis suggests that various neuropsychiatric clusters and symptoms are associated with impairment in overall and specific cognitive abilities. The main hypothesis in this review was that there would be a negative association between NPS and cognition, i.e. that experiencing the NPS (as opposed to not experiencing it) or experiencing it at a higher frequency or severity is associated with worse cognition. All significant associations found in this review were negative, thus confirming our hypothesis.

Specifically, a negative association was found between overall NPS and overall cognition, which is consistent with previous studies that suggested that NPS were linked to worse cognition in people with mild cognitive impairment (MCI) and dementia, faster cognitive decline and progression from MCI to dementia (Chan et al., 2011; Dietlin et al., 2019; Teng et al., 2007).

The affect cluster was shown to be linked with impairments in global cognition, as well as in attention and semantic knowledge. Within this cluster, apathy was most frequently associated with cognition, including with global cognition and with various cognitive domains. Again, this is consistent with previous research that suggested that apathy was associated with worse cognition and that it is predictive of conversion from MCI to dementia (Fresnais et al., 2022).

No statistically significant associations were found between the executive dysfunction cluster or disinhibition and cognition in this review. A possible explanation for this is that only a few studies (between 2 and 9) investigated these relationships and therefore a significant association could not be found. The same was true for social inappropriateness since there were no studies investigating the relationships between this symptom and cognition.

The aggression and motor disturbances clusters were also found to be associated with global cognition. An association was found between agitation and worse global cognitive performance.

Psychotic symptoms were linked with worse overall cognition and impairment in some cognitive domains, such as executive function and working memory. Within this cluster, hallucinations only showed an association with global cognition, while delusions were found to be associated with several cognitive domains.

Few studies (between 3 and 9) investigated the relationship between circadian rhythms and cognition in dementia, limiting the evidence in this domain, and no significant associations were found in this review in these domains.

This is the first systematic review to synthesise the existing evidence regarding the specific associations between NPS and cognition. Our methodology was robust, including a broad search strategy, which resulted in the retrieval of thousands of items for screening, and all steps of the review process were conducted independently by two or more reviewers.

This study demonstrates that there is an association between overall NPS symptoms and worse cognitive performance, and between specific NPS and worse performance in specific cognitive domains. Despite the large number of studies included in this review, there is still relatively little research looking at the associations between some NPS, particularly those in the executive dysfunction and circadian rhythms clusters, and cognition in people living with dementia. Furthermore, due to the significant heterogeneity that characterised the associations, the relationships we found require further investigation to confirm their validity. This might include the use of standardised measures for various NPS and cognitive domains. Additionally, synthesis of longitudinal results is warranted to detect whether NPS precede or are a consequence of cognitive impairment or whether there is a bidirectional or more complex relationship between them. Moreover, future studies should investigate whether the nature of some of the identified relationships changes over time, depending on the stage of the disease, or following interventions and other treatments.

The aetiology behind NPS is known to be complex and multidimensional (Kálmán et al., 2008), likely with biological, psychological, social and cognitive contributions. This systematic review looked solely at the potential cognitive aspect of this aetiology. However, while this may be considered a limitation, our findings suggest that if we can manage the cognitive deficits using alternative strategies such as biological, psychological and social treatment approaches, we will also address some of the other facets that contribute to the expression of NPS in people with dementia.

Several other limitations warrant consideration in the interpretation of the results of this review. First, for practical reasons, studies for which we were not able to obtain a manuscript in English were excluded. Furthermore, studies that did not present data as Pearson/Spearman correlations or mean differences and for which it was not possible to obtain the data from the authors were excluded, which lessened the scope of our review. However, given the large number of included studies, these exclusions are unlikely to have biased the results. Additionally, due to the large number of studies that were included and practical constraints, a decision was made to focus on the associations between NPS and objective cognition, excluding the investigation of subjective cognition (e.g. perception of one’s memory ability). Moreover, cognitive and NPS were clustered using a classification system created by the authors of this review to account for the lack of a gold standard classification system that could accommodate the various domains reported by the primary studies. We acknowledge that different results may have been found if a different system for clustering domains had been followed. However, the current classification system was developed following the clinical and research expertise of the authors of the review in the fields of neuropsychology and neuropsychiatry.

A further limitation of the review is that the majority of studies focused on people with dementia due to AD and, in most cases, there were not enough data for people with other dementia diagnoses (e.g. DLB or PD). While previous research has shown that different patterns of NPS can be found in different types of dementia (Majer et al., 2019), the relatively little body of work of the associations between NPS and cognition in people with dementia diagnoses other than AD made it difficult to explore whether the relationships are different for people with different types of dementia. Future research should aim to expand the study of these associations to people with a wide range of dementia diagnoses including dementia due to mixed pathologies. It was also not possible to investigate other potential moderators of the identified associations (e.g. age at onset of dementia or severity of dementia) due to poor reporting of these characteristics.

Other potential causes of bias include the heterogeneity between studies in relation to the scales used to measure NPS and tests used to measure cognition and the varying severity of dementia. Although it was not possible to investigate whether the latter moderates the relationships due to insufficient data about participants’ dementia severity, the associations found in our review differ from those found by a recent cross-sectional study that included 7179 cognitively unimpaired older adults (Liampas et al., 2022). For example, Liampas and colleagues found associations between anxiety and impairment in semantic memory and between hallucinations and worse executive functions, both of which were not found in our review of people with dementia. While some of these differences are likely driven by methodological factors, a possible explanation is that the associations between NPS and cognition are not stable throughout the trajectories of cognitive decline. This raises the question of whether the underlying neuroanatomical changes of people with dementia (Wang et al., 2003), that are not present in cognitively unimpaired people, play a role in mediating these associations. While this was beyond the scope of the current review, future studies should explore the associations between NPS and cognition in light of the recent developments in the field of the neurobiological underpinnings of both kinds of symptoms. Furthermore, the construct of dementia itself has suffered several modifications throughout the years, with a shift from diagnostic criteria based primarily on symptomatology towards an approach that includes results from imaging and biomarkers tests. This, coupled with the evolution in the way cognition and NPS are understood and operationalised, could further interfere with the correct interpretation of the associations found. Future reviews should address this heterogeneity controlling for these or other variables. A better understanding of these issues could lead to improvement in early treatment of such symptoms and therefore allow for better clinical outcomes, as well as carer burden relief for family and professional caregivers, and saving of costs associated with care of the person with dementia.

This review demonstrates a link between certain NPS and impairment in specific cognitive skills in people with dementia. Of course, the associations found in the current study do not establish causality. It is not possible to determine, based on results from this meta-analysis, whether NPS precede, result from, or simply coexist with cognitive impairment, or whether there is a complex relationship with overlapping pathologies, interaction with the environment and other health factors and varying depending on the stage of the disease. In any case, these findings should incentivise researchers to further examine co-occurrence of these symptoms and explore novel treatment approaches that target both kinds of symptoms simultaneously, as well as aim to better understand the nature of the relationship between them.

### Supplementary Information

Below is the link to the electronic supplementary material.

Supplementary file1 (DOCX 23 KB)

Supplementary file2 (DOCX 70 KB)

Supplementary file3 (DOCX 38 KB)

Supplementary file4 (DOCX 93 KB)

Supplementary file5 (DOCX 42 KB)

## Data Availability

All relevant data are presented in this manuscript and its supporting files.
