# Supplementary material for: The Associations Between Neuropsychiatric Symptoms and Cognition in People with Dementia: A Systematic Review and Meta-Analysis
Source: Neuropsychol Rev. 2023 Jul 21;34(2):581–97. doi: 10.1007/s11065-023-09608-0 (PMC11166771; doi:10.1007/s11065-023-09608-0)
Supplement: Supplementary file 1 — Supplementary file1 (DOCX 23 KB) [file 11065_2023_9608_MOESM1_ESM.docx]

**The associations between neuropsychiatric symptoms and cognition in people with dementia: A systematic review and meta-analysis**

*Neuropsychology review*

Ms. Julieta Sabates, The University of Melbourne, Australia.

Ms. Wei-Hsuan Chiu, The University of Melbourne, Australia.

A/Prof Samantha Loi, The University of Melbourne, Royal Melbourne Hospital, Australia.

Dr. Amit Lampit, The University of Melbourne, Australia.

Dr. Hanna M Gavelin, The University of Melbourne, Australia; Department of Psychology, Umea University, Sweden.

Dr. Terence Chong, The University of Melbourne, St Vincent’s Hospital Melbourne, Royal Melbourne Hospital, Australia.

Ms. Nathalie Launder, The University of Melbourne, Australia.

Dr.Anita MY Goh, National Ageing Research Institute; The University of Melbourne, Australia.

Prof. Amy Brodtmann, Cognitive Health Initiative, Central Clinical School, Monash University, Australia.

Prof. Nicola Lautenschlager, The University of Melbourne, Australia.

A/Prof. Alex Bahar-Fuchs, The University of Melbourne, Australia.

Corresponding author: Ms Julieta Sabates. Mailing address: 151 Barry Street, Carlton 3053, Victoria, Australia; Email address: Julieta.sabates@unimelb.edu.au

Supplementary material: S1- Search strategy

1 (Dement* or Alzheim* or AD or Frontotemporal or FTD or Neurocognitive disorder* or Parkinson* or Huntington or Lewy Bod*).ab,ti.

2 (Neuropsychiatr* or BPSD or Behavio* or psycholog* or Apath* or depress* or Anxi* or Aggress* or Agitat* or Aberrant motor or Irritab* or disinhibit* or eat* disturbance* or eat* problem* or sleep* disturbance* or psychotic or psychosis or hallucinat* or delu* or wander* or impuls* or mood).ab,ti.

3 (neuropsycholog* or cognition or cognitive function* or cognitive skill* or cognitive abilit* or cognitive impairment* or cognitive dysfunction* or cognitive problem* or cognitive profile* or cognitive deficit* or memory or Attention* or Executive or speed or language or visuospatial or visuoconstruction* or fluency or learning).ab,ti.

4 2 adj6 3

5 (associat* or relat* or correlat*).ab,ti.

6 4 adj6 5

7 1 and 6
