# Supplementary material for: The Associations Between Neuropsychiatric Symptoms and Cognition in People with Dementia: A Systematic Review and Meta-Analysis
Source: Neuropsychol Rev. 2023 Jul 21;34(2):581–97. doi: 10.1007/s11065-023-09608-0 (PMC11166771; doi:10.1007/s11065-023-09608-0)
Supplement: Supplementary file 2 — Supplementary file2 (DOCX 70 KB) [file 11065_2023_9608_MOESM2_ESM.docx]

**The associations between neuropsychiatric symptoms and cognition in people with dementia: A systematic review and meta-analysis**

*Neuropsychology review*

Ms. Julieta Sabates, The University of Melbourne, Australia.

Ms. Wei-Hsuan Chiu, The University of Melbourne, Australia.

A/Prof Samantha Loi, The University of Melbourne, Royal Melbourne Hospital, Australia.

Dr. Amit Lampit, The University of Melbourne, Australia.

Dr. Hanna M Gavelin, The University of Melbourne, Australia; Department of Psychology, Umea University, Sweden.

Dr. Terence Chong, The University of Melbourne, St Vincent’s Hospital Melbourne, Royal Melbourne Hospital, Australia.

Ms. Nathalie Launder, The University of Melbourne, Australia.

Dr.Anita MY Goh, National Ageing Research Institute; The University of Melbourne, Australia.

Prof. Amy Brodtmann, Cognitive Health Initiative, Central Clinical School, Monash University, Australia.

Prof. Nicola Lautenschlager, The University of Melbourne, Australia.

A/Prof. Alex Bahar-Fuchs, The University of Melbourne, Australia.

Corresponding author: Ms Julieta Sabates. Mailing address: 151 Barry Street, Carlton 3053, Victoria, Australia; Email address: Julieta.sabates@unimelb.edu.au

Supplementary material: S2- Expanded table of characteristics of included studies

| **Study ID** | **Study** | **Sample size (Dementia)** | **Aim** | **Gender ratio (Dementia; % female)** | **Ethnicity** | **Young vs Late onset / Age of onset** | **Dementia syndrome** | **Severity (CDR) / Global cognition (MMSE)** | **Hospitalisation** | **Medications** | **Notes** | **NPS measures used** | **Cognitive measures used** |
| --- | --- | --- | --- | --- | --- | --- | --- | --- | --- | --- | --- | --- | --- |
| Akyol 2020 | Cross-sectional | 106 | To compare apathy across three types of dementia and determine the factors affecting apathy for each type. | 46.23% | NR | NR | AD, VaD, FTD | MMSE = 15.77 | NR | No antipsychotics |  | NPI | MMSE |
| Balci 2011 | Cross-sectional | 26 | To examine the relationship between cognition, physical status and depression. | 80.80% | NR | NR | AD | MMSE = 15.69 | NR | NR |  | Yesavage Geriatric Depression Scale | MMSE, Stroop (Naming ink colours) |
| Bhat 2021 | Cross-sectional | 76 | To explore the behavioral dysfunction vascular dementia and compare the impairment in various behavioral domains. | 28.90% | NR | NR | VaD | CDR = 1.65 | NR | NR |  | NPI | Frontal Assessment Battery |
| Benedict 1999 | Case-control | 13 | To investigate how fluctuations in mood states interact with cognitive performance in a diverse sample of psychiatry inpatients. | 92.00% | All Caucasian | NR | VaD | MMSE = 22.4 | Yes | 87% of the sample |  | Montgomery-Asberg Depression Rating Scale | MMSE, BNT, Visual-Motor Integration, HVLT-R, BVMT-R, TMT B |
| Breitve 2018 | Longitudinal | 196 | To investigate if apathy is linked with shorter survival and higher risk of nursing home admission, and if apathy is associated with cognitive impairment at baseline. To examine if apathy is associated with faster global cognitive decline. | 37.00% | NR | NR | AD, DLB | CDR = 0.87 | NR | AD non-apathy = 51.9%; AD apathy = 41.2%; DLB non-apathy = 54.8%; DLB apathy = 30.3% |  | NPI-Apathy | MMSE, CVLT-II, VOSP, BNT, Stroop test, COWAT, TMT A |
| Bronnick 2011 | Cross-sectional | 172 | To investigate the cognitive profile in parkinson's disease patients with visual hallucinations as compared to those without. | 38.00% | NR | NR | PDD | MMSE = 20.11 | NR | No antipsychotic medication |  | NPI | MMSE, Simple reaction time, Go/no-go reaction time, Choice reaction time, Serial sevens reversed, Digit vigilance, Letter fluency, Word recognition, Word recall, Commands, Constructional praxis, Naming objects/finger, Ideational praxis |
| Bylsma 1994 | Longitudinal | 180 | To examine the effects of extrapyramidal symptoms, psychopathological symotoms, and myoclonus on the rate of cognitive and functional decline in AD. | 58.00% | NR | Late(68.35) | AD | CDR = 1.2 | NR | No antipsychotic medication |  | Columbia University Scale for Psychopathology in Alzheimer's Disease - Delusions | MMSE, Naming |
| Camargo 2017 | Cross-sectional | 40 | To analyse apathy and depression in patients with PDD. | 35.00% | NR | NR | PDD | SCOPA-Cog = 11.25 | NR | Levodopa therapy |  | Montgomery-Asberg Depression Rating Scale, Apathy Evaluation Scale | SCOPA-Cog |
| Chwiszczuk 2017 | Longitudinal | 246 | To determine if patients with mild dementia with and without REM sleep behaviour disorder differ in progression rate and in specific neuropsychological measures. | 56.56% | NR | NR | AD, DLB, PDD | MMSE = 23.64 | NR | Some participants Parkinson's medication and dementia medication |  | Mayo Sleep Questionnaire | MMSE |
| Contador-Castillo 2009 | Case-control | 23 | To compare the cognitive performance of patients with AD with and without depression, patients with major depressive disorder, and a control group. | 65.22% | NR | NR | AD | MMSE = 22.73 | NR | NR |  | DSM-IV-TR | MMSE, Clock drawing test, MDRS, Direct digits, Inverse digits, Word free recall, Word recognition, Position free recall, Drawing recognition |
| D'antonio 2019 | Longitudinal | 32 | To investigate (a) whether an impairment in specific cognitive domains predicts the onset of psychosis in AD patients and (b) what grey matter alterations, their location, and the rate of atrophy differentiate AD patients with and without psychosis. | 50.00% | NR | NR | AD | MMSE = 19.63 | NR | NR |  | NPI-Psychosis | MMSE, RAVLT, Babcok test, Digit span, Corsi block tapping test, RCFT, Visual search, TMT A, TMT B, Verbal fluency, BNT, Clock drawing test, Frontal Assessment Battery, Raven’s progressive coloured matrices |
| de Oliveira 2015 ("Correlations…") | Cross-sectional | 217 | 1) To verify correlations among assessments for cognition, behaviour and functional independence in patients with AD. 2) To estimate impacts of education, APOE haplotypes, length of dementia, age and alcohol use over the neuropsychiatric assessment. | 67.70% | NR | Late (73.19) | AD | CDR 1, 2, 3 | NR | NR |  | NPI | MMSE, Clock drawing test |
| de Oliveira 2015 2 ("Contrasts...") | Cross-sectional | 39 | To compare and distinguish demographic and neuropsychiatric features between LBD and APOEE3/E3 late-onset AD. | 56.41% | NR | Late | AD, PDD, LBD | MMSE = 17.52 | NR | NR |  | NPI | MMSE, Clock drawing test, Digit span |
| de Oliveira 2020 | Cross-sectional | 51 | To evaluate risk factors for age at and which neuropsychiatric features are associated with pharmacotherapy and signs and symptoms of LBD. | 39.20% | NR | NR | PD, LBD | MMSE = 15.82, CDR = 11.46 | NR | 76.5% cholinesterase inhibitors, 7.8% memantine, 47.06% Levodopa, 47.06% anti-psychotic, 58.82% anti-depressants |  | NPI | MMSE, Clock drawing test, Digit span |
| Demichele-Sweet 2011 | Longitudinal | 2317 | To more reliably determine the correlates of the AD+Psychosis syndrome. To look at the association of APOE 4 with AD+Psychosis. | 57.00% | 84.5% Caucasian, 12% African American, 3.5% other | Late (72.3) | AD | MMSE 17.7 | NR | NR |  | NPI-Psychosis | MMSE |
| de Paula 2016 | Cross-sectional | 93 | To evaluate how depressive symptoms moderate cognitive and functional performance in people with AD. | NR | NR | NR | AD | MMSE = 20.59 | NR | NR |  | Geriatric Depression Scale-15 | Language semantic, Episodic memory, Visuospatial abilities, Executive functions |
| Drijgers 2011 | Cross-sectional | 260 | To explore the association between apathy and neuropsychological functioning in patients with MCI and AD. | 56.00% | NR | NR | AD | MMSE 20.3 | NR | NR |  | NPI-Apathy | MMSE, Verbal learning test, Stroop, Concept shifting task, Verbal fluency |
| Eikelboom 2021 | Cross-sectional | 1090 | To investigate the prevalence and course of NPS, and associations between baseline NPS and performance on cognitive domains at baseline and over time in an amyloid-β positive sample. | 52.40% | NR | NR | AD | MMSE = 20.3 | NR | NR |  | NPI | MMSE, RAVLT, Visual association test A, Digit span, Stroop, Frontal Assessment Battery, Category fluency, Naming, VOSP |
| Eustace 2001 | Cross-sectional | 150 | To determine the prevalence of verbal aggression and identify associated clinical, functional and neuropsychological correlates. | 69.00% | NR | NR | AD | MMSE = 19.31 | NR | NR |  | BEHAVE-AD | MMSE |
| Fahlander 1999 | Longitudinal | 54 | To investigate if depression exacerbates the memory deficits associated with AD. | 76.00% | NR | NR | AD | MMSE 19.57 | NR | NR |  | DSM-III-R | MMSE |
| Fernandez 2010 | Cross-sectional | 1014 | To describe the NPS and the clinical profile of AD patients showing lower and higher behavioural symptoms. To evaluate the relationship between NPS and cognitive impairment severity. | 65.00% | NR | NR | AD | NR | NR | 94% antidementia drugs, 37% antipsychotic drugs. |  | ADAS-Noncog | MMSE |
| Fernandez Martinez 2010 | Cross-sectional | 99 | To study the prevalence of NPS in patients with AD, amnestic MCI and controls, their relationship to dementia severity; and to explore the relationship between the NPS with specific neuropsychological tests. | 56.60% | NR | NR | AD | MMSE = 21.93 | NR | No antidementia or psychotropic medications |  | NPI | MMSE |
| Fillit 2021 | Cross-sectional | 6265 | To compare characteristics and clinical outcomes among long‐term care residents with dementia with and without agitation. | 69.00% | 64.9% White, not of Hispanic origin; 16.9% Black, not of Hispanic origin; 16.6% Hispanic; 1.18% Asian/Pacific Islander; 0.42% American Indian/Alaskan | NR | AD and other aetiologies | NR | All long-term care residents | 47.61% antipsychotics; 62.69% antidepressants; 51.20% antidementia drugs; 37.46% antianxiety drugs; 41.64% narcotics; 37.48% antiepileptics | Secondary analysis | Agitation Minimum Data Set | Brief Interview for Mental Status |
| Fitz 1994 | Cross-sectional | 91 | To investigate the link between depression and functional and cognitive impairment in patients with AD. | 55.00% | NR | NR | AD | MDRS = 102.52 | NR | NR |  | Hamilton Depression Rating Scale | Attention, Initiation/perseveration, Construction, Conceptualization, Memory |
| Flynn 1991 | Cross-sectional | 33 | To investigate if delusions are a marker for subtypes of the disease process, and their relationship to specific intellectual alterations in dementias. To explore differences between the delusions of AD and MID. | 9.00% | NR | NR | AD / MID | MMSE = 16.5 | NR | 6 neuroleptic treatment |  | BEHAVE-AD Delusions | MMSE, Memory, BNT, Verbal fluency, Visual-motor integration test, Abstraction |
| Gallassi 2001 | Case-control | 33 | To investigate the comorbidity between depression and dementia. | 29.00% | NR | NR | AD | MMSE = 15.4 | NR | NR |  | DSM-IV | MMSE |
| Gallo 2008 | Cross-sectional | 48 | To investigate the degree to which BPSD and neurocognitive function are associated with functional independence among outpatients with mild dementia. | 65.00% | NR | NR | AD, VaD | MMSE = 23 | No | NR |  | NPI | MMSE, Phonetic fluency, Clock drawing test, Boston naming test, Wechsler Adult Intelligence Scale - Revised Similarities, Delayed free recall, Long-term recognition trial |
| Galynker 1995 | Cross-sectional | 26 | To examine the relationship of negative symptoms in AD to both depressive symptoms and cognitive impairment. | 57.70% | NR | NR | AD / Multi-infarct dementia | MMSE = 16.8 | NR | 7 antipsychotic, 6 benzodiazepines, 4 antidepressants |  | Positive and Negative Syndrome Scale, Hamilton Rating Scale for Depression | MMSE |
| Gilley 1991 | Cross-sectional | 230 | To evaluate the clinical significance of hallucinations in AD. | 67.00% | 85.65% White, 14.35% Non-White | NR | AD | MMSE 12.57 | No | No psychotropic medication |  | A standardized structured interview | MMSE |
| Grossi 2013 | Cross-sectional | 61 | To explore the neuropsychological correlates of apathy in patients with PDD and AD, and to identify the cognitive profile of apathy in the two typs of dementia. | 46.00% | NR | NR | AD, PDD | MMSE = 22.13 | NR | NR |  | Informant and Self-rated versions of the Apathy Evaluation Scale | MMSE, Frontal Assessment Battery, Clow drawing test, Corsi’s block tapping test, Verbal span for bysillabic words, Immediate recall, Delayed recall, Raven’s Coloured Progressive Matrices, Apraxia constructional task, ROCF, Stroop test, TMT, Verbal fluency, Inverse motor learning test |
| Hallikainen 2012 | Longitudinal | 236 | To examine if global cognitive performance is associated with dementia severity, activities of daily living and NPS in patients with very mild or mild AD. | 51.30% | NR | NR | AD | MMSE = 21.5 | NR | 96.2% AD medication |  | NPI | MMSE |
| Harwood 2000 | Cross-sectional | 114 | To examine the relationship of BPSD to cognitive and functional impairment in AD. | 63.00% | White or non-Hispanic 56%; Hispanic 44% | NR | AD | MMSE = 17.8 | NR | NR |  | BEHAVE-AD, Revised Memory and Behaviour Problems Checklist | MMSE |
| Hopkins 2005 | Cross-sectional | 48 | To explore whether psychotic symptoms in patients with dementia might are due to greater executive control and visuoperceptual deficits. | NR | NR | NR | VaD | MMSE = 21/33 | No | No psychoactive medication |  | NPI | MMSE, COWAT, Clock drawing test, BNT, Word list generation, CVLT |
| Ito 2007 | Longitudinal | 40 | To assess the possible neurological basis of BPSD. | 68.00% | NR | NR | AD | CASI = 25.15 | All patients | 13 patients in the risperidone group, and 15 patients in the non-risperidone group continued to receive 5 mg/day of donepezil. |  | BEHAVE-AD | Cognitive Abilities Screening Instrument |
| Janzing 2005 | Cross-sectional | 60 | To investigate to what extent depression and cognitive dysfunction are related in people with dementia. | 88.30% | NR | NR | NR | NR | NR | NR |  | DMS-III-R | Verbal fluency Animals, Modified BNT, Word list memory, constructional praxis, Word list recall, word list recognition |
| Keator 2019 | Cross-sectional | 58 | To investigate correlations between language and behavioral scores across three variants of PPA. | 50.00% | NR | NR | PPA | NR | No | NR |  | Frontal Behavioural Inventory | Semantic word picture matching, Pyramids and Palm Trees Test, Sentence repetition, Hopking Assessment of naming actions, BNT |
| Kuzis 1999 | Cross-sectional | 184 | To investigate the association between apathy and depression, and specific cognitive deficits in AD. | NR | NR | NR | AD | MMSE = 22.49 | NR | NR |  | Hamilton Depression Scale | MMSE, Buschke recall, BNT, Wisconsin Card Sorting Test, Verbal fluency, Raven’s Progressive Matrices, Purdue Pegboard Test, Block design, Similarities, Benton Visual Retention Test, Token Test, Digit span |
| Kwak 2013 | Cross-sectional | 230 | To address the prevalence and the associated factors of delusion subtypes in Korean patients with drug-naïve probable AD, and compare the clinical characteristics of each delusion subtype. | 61.73% | Asian (Korean) | Late (71.88) | AD | CDR = 1.06 | NR | No psychotropic medication |  | NPI-Delusions | MMSE |
| Lam 2006 | Case-control | 125 | To assess the clinical correlates of challenging NPS using the Chinese version of the Challenging Behaviour Scale (CCBS) designed for residential care settings. | 58.40% | All Chinese | NR | AD, VaD | MMSE = 8.62 | NR | 40.8% antipsychotics, 32.8% antidepressants, 16.8% hypnotic, 6.4% antidementia drugs. |  | Challenging Behaviour Scale | MMSE |
| Lee 2007 | Case-control | 50 | To investigate the relationship between confabulation and delusion and to clarify the mechanism of confabulation in AD. | 86.00% | Asian (Japanese) | NR | AD | MMSE = 15.58 | NR | 39 donepezil; 11 antipsychotic drugs |  | Semantic Confabulations | MMSE |
| Lee 2012 | Cross-sectional | 127 | To explore the specific subgroups of NPS and investigate the possible correlation between cognitive functioning and NPS in PDD patients. | 40.15% | NR | NR | PDD | MMSE = 17 | NR | NR |  | NPI | MMSE |
| Lee 2019 | Cross-sectional | 1247 | To investigate the differential association between depression and cognitive function in patients with MCI and AD. | 56.60% | Asian (Korean) | NR | AD | MMSE = 20.21 | NR | NR |  | Geriatric Depression Scale | Attention, Language, Visuospatial, Memory, Executive |
| Levy 1998 | Cross-sectional | 28 | To explore the relationship of apathy and depression, and to examine the relationship of these symptoms to cognitive impairment. | 46.43% | NR | NR | AD, FTD | MMSE = 16.5 | NR | NR |  | NPI-apathy, NPI-depression | MMSE |
| Logsdon 1998 | Longitudinal | 193 | To investigate the frequency of wandering behavior in people with AD. To explore demographic, cognitive, functional, and behavioral differences between wanderers and nonwanderers, and to identify predictors of wandering. | 49.00% | 89% Caucasian, 7% African American, 3% Asian/Pacific Islanders | NR | AD | MMSE = 17.4 | No | NR |  | Wandering ad hoc scale | MMSE |
| Lopez 1991 | Longitudinal | 17 | To evaluate the neuropsychological and neurophysiologic characteristics of AD patients with and without delusions and hallucinations. | 76.00% | NR | NR | AD | MMS = 18.7 | No | 6 patients with psychotropic medication |  | DSM-III-R | Speed/Attention, Memory/Learning, Expressive language, Receptive language, Visuospatial |
| Machado 2020 | Cross-sectional | 32 | To analyse associations of neuropsychiatric and motor assessments with language and visual organisation in patients with DLB. | 59.00% | NR | AOO = 71.14 | DLB | MMSE = 17.72 | NR | Daily amount of different medications = 5.03 |  | NPI | MMSE, Verbal fluency, BNT, Clock drawing test, Digit span, Hooper |
| Mariano 2020 (Guimaraes 2020) | Case-control | 42 | To explore if there is an association between apathy and social cognition. | 45.23% | NR | YOD (64.08) | AD, FTD | MMSE = 25.06 | NR | NR |  | Apathy Scale | MMSE, Digit span, Frontal Assessment Battery, Verbal fluency, Figure memory test, Facial emotion recognition test, Faux-Pas Test, Mini-SEA |
| Mc Pherson 2002 | Cross-sectional | 44 | To explore the associations between apathy, depression and executive ability in patients with AD. | 69.00% | NR | NR | AD | MMSE = 22.57 | NR | NR |  | NPI-Apathy | MMSE, Wechsler Adult Intelligence Scale-Revised, Verbal fluency, BNT, ROCF, Finger tapping test, TMT-B, Stroop |
| Migliorelli 1995 | Cross-sectional | 103 | To investigate the prevalence and type of delusions in AD, and to examine the neurological, neuropsychological and psychiatric correlates. | 74.00% | NR | Late (69.21) | AD | NR | NR | 9 patients (8.74%) = on tricyclic drugs, 2 patients (1.94%) = on neuroleptics, 23 patients (22.33%) = on benzodiazepines |  | Dementia Psychosis Scale | MMSE, Buschke, Token test, BNT, Wisconsin Card Sorting Test, TMT, COWAT, Raven’s progressive matrices, Digit span, Block design, Analogies |
| Mizrahi 2006 | Cross-sectional | 771 | To explore the frequency and clinical correlates of paranoid and expansive delusions in AD, and to assess the validity of the different dignostic criteria for psychosis in AD. To assess if anosognosia and depression are significant and independent predictors of psychosis in AD. | NR | NR | NR | AD | MMSE = 21.8 | NR | NR |  | Dementia Psychosis Scale, DSM-IV | MMSE |
| Montagnese 2021 | Cross-sectional | 284 | To study the links between cognition, hallucination-specific insight, temporal, emotional and severity aspects of hallucinations and what aspects of the links might differ when comparing neurodegenerative disease and eye disease. | 49.00% | NR | NR | DLB, PDD | MMSE = 26.05 | NR | NR |  | Hallucinations Severity and Frequency (Measure unclear) | MMSE |
| Na 2017 | Cross-sectional | 289 | To demonstrate the relationship between delusion of theft and cognitive functions in mild AD. | 71.00% | NR | NR | AD | CDR = 1, MMSE = 20.83 | NR | NR |  | Delusions (Measure unclear) | MMSE, Verbal fluency, BNT, Word list memory, constructional praxis, Word list, Constructional recall |
| Naarding 2006 | Case-control | 54 | To investigate if depressive symptoms in dementia reflect dysfunction in fronto-subcortical pathways. | 46.00% | NR | NR | Post-stroke dementia | MMSE = 19.8 | Yes | NR |  | Schedule for Affective Disorders and Schizophrenia | MMSE, Word list recall, Verbal fluency |
| Nagata 2010 | Cross-sectional | 50 | To study the association between frontal lobe function, including executive function, and activity disturbances. | 78.00% | NR | NR | AD | MMSE = 19.38 | NR | NR |  | BEHAVE-AD | Frontal Assessment Battery |
| Nagata 2017 | Cross-sectional | 421 | To analyse the links between each symptomatic cluster and psychosocial and clinico-demographic factors in people with AD who needed treatment for psychotic symptoms or agitation. | 56.00% | 79% Caucasian | NR | AD | MMSE = 15 | No | 47.5% anti-dementia drugs, 21.9% antidepressants, antipsychotics or anxiolytics/hypnotics. |  | NPI-aggression, NPI-psychosis | MMSE |
| Nakaaki 2007 | Cross-sectional | 42 | To explore the wide range of deficits of executive function in AD patients with and without depression. | 52.38% | All Japanese | NR | AD | MMSE = 19.55 | NR | No acetylcholine esterase inhibitors or antidepressants. |  | Hamilton Rating Scale for Depression (HRSD) + DSM-IV-TR | MMSE, WAIS-R, ROCF, RAVLT, WMS-R, Letter fluency, Stroop task, TMT, Digit symbol, Digit span, Boxes filled number, Dual-task condition |
| Nakaaki 2008 | Cross-sectional | 88 | To investigate if AD patients with apathy and depression have greater deficits in their Frontal Assessment Battery and dual task scores. To investigate the link between these two symptoms and the performance of the subtests included in the battery. | 55.00% | NR | NR | AD | MMSE = 19.76 | No | No cholinesterase inhibitors or antidepressants. |  | NPI-Apathy | MMSE, Frontal Assessment Battery |
| Nakatsuka 2014 | Cross-sectional | 142 | To tstudy if cognitive decline in AD specifically correlates with some particular types of delusional thoughts. | 66.90% | NR | NR | AD | MMSE 14.4 | NR | None |  | BEHAVE-AD - Delusions | MMSE |
| Onyike 2007 | Longitudinal | 316 | To investigate the distribution of apathy in older adults and its links with cognition and daily functioning. | NR | NR | NR | NR | MMSE = 14.32 | No | NR |  | NPI-Apathy | MMSE, Word list recall, BNT, BVRT, Contructional praxis, Animal fluency, COWAT, Symbol-digit modality, TMT |
| Pagonabarraga 2008 | Cross-sectional | 30 | To explore delusional misidentification syndromes in PDD by describing their prevalence and nature in a cohort of 30 PDD patients and detailing their neuropsychological correlates with a comprehensive neuropsychological battery. | 43.00% | NR | NR | PDD | MDRS = 102.33 | NR | Levodopa; dopaminergic agonists-LD equivalent. | They used the Delusional misidentification syndromes (NPS measure), designed by the authors of the study | The delusional misidentification syndromes, defined by the study | Digit span, RAVLT, Verbal fluency, BNT, Judgement of line orientation test, Poppelreuter-Ghent |
| Park 2019 | Cross-sectional | 1128 | To examine the factors influencing BPSD in AD patients using data from the Clinical Research of Dementia of South Korea (CREDOS) study. | 62.00% | Asian (Korean) | NR | AD | MMSE = 20.1 | NR | NR |  | NPI | MMSE |
| Perneczky 2009 | Case-control | 21 | To determine the association between structural pathology and delusion symptomatology in DLB. | 48.00% | NR | Young (63.1) | DLB | MMSE = 20.8 | NR | Levodopa |  | NPI-Delusions | MMSE |
| Perri 2014 | Cross-sectional | 86 | To identify the neuropsychological correlates of BPSD in patients with various forms of dementia. | 39.53% | NR | NR | AD, frontal variant FTD, SIVD, LBD | CDR = 0.72, MMSE = 22.53 | NR | No anticholinesterase or neuroleptic medications. |  | NPI-Apathy, NPI-Hallucinations | Modified Card Sorting Test, Copy of drawings test |
| Perri 2018 | Cross-sectional | 20 | To study the link between cognitive, emotional-affective and auto-activation apathy and performance on tasks investigating executive and theory of mind functions in AD. | 55.00% | NR | NR | AD | MMSE = 22.2 | NR | If antidepressant or anticholinesterasic medication was being taken, it had to be stable for the last six months. Patients under neuroleptic or antiepileptic therapy were excluded. |  | Diary (Apathy) | Verbal fluency, Digit span backward, Corsi span backward, TMT, Modified Card Sorting Test, First-order False belief task, Emotion attribution, Emotion recognition |
| Pezzoli 2019 | Case-control | 52 | To explore the neuroanatomical, and neuropsychological characteristics related to visual hallucinations in DLB and Parkinson's disease without dementia. | 46.00% | NR | NR | DLB | MMSE = 25.42 | NR | Medications used included: cholinesterase inhibitors and/or levodopa, benzodiazepines, antidepressants, dopamine agonists, and monoamine oxidase inhibitors. |  | NPI-Hallucinations | MMSE, Digit span, Immediate recall, Delayed recall, Phonemic fluency, Clock drawing test, Rey figure recall, VOSP, Digit cancellation, TMT, ROFC, VOSP Silhouettes |
| Qian 2018 | Longitudinal | 900 | To study the links between psychosis and APOE ε4 allele and cognitive and functional outcomes. To explore the links between APOE ε4, Lewy bodies, and psychosis. | 44.00% | NR | Late | AD | MMSE = 13.17 | NR | NR |  | NPI-Psychosis | MMSE |
| Quaranta 2015 | Cross-sectional | 108 | To analyse the relationship between psychotic symptoms and cognitive impairment in AD. | 64.00% | NR | NR | AD | MMSE = 17.2 | NR | No antipsychotic medication. |  | NPI-Hallucinations | MMSE, RAVLT, ROCF, Raven’s progressive matrices, Immediate visual memory, Digit span, Spatial span, Verbal fluency, Object naming, Copy of figures, Stroop test |
| Reed 1993 | Cross-sectional | 57 | To investigate if depression and anosognosia are related to each other and to overall dementia severity, and if anosognosia is linked with regional cerebral function in AD. | NR | NR | NR | AD | MMSE = 19.4 | NR | No psychotropic medication. |  | DSM-III | MMSE |
| Rochat 2013 | Case-control | 30 | To compare patients with mild AD and matched controls on four dimensions of impulsivity. To study the link between impulsivity changes and cognitive performances on executive/attentional tasks in mild AD and healthy controls. | NR | NR | NR | AD | NR | NR | NR |  | UPPS – Impulsive Behaviour Scale | MMSE, Go/No-go, Letter number sequencing |
| Rolland 2007 | Longitudinal | 682 | To explore the predictive value of wandering behavior at baseline for nutritional status, disability, institutionalisation, and mortality at 2 years of follow-up. | 72.00% | NR | Late (74.1) | AD | MMSE = 20.1 | NR | NR |  | NPI-Aberrant motor behaviour | MMSE |
| Ross 1998 | Cross-sectional | 1486 | To examine the relationship between cognitive and functional impairment in depressed and non-depressed people with AD. | 69.00% | 75.68% Caucasian, 8.92% African-American, 10.01% Hispanic, 5.38% Asian/Filipino/Pacific Islander | NR | AD | MMSE = 16.62 | No | NR |  | Diagnosis of depression by physicians and neuropsychologists | MMSE |
| Rozum 2019 | Longitudinal | 56 | To investigate the prevalence of NPS and cognitive correlates in severe dementia. | 67.90% | NR | NR | VaD | SCIP total = 154.77 | NR | NR |  | NPI | Severe cognitive Impairment Profile |
| Ruiz 2018 | Cross-sectional | 90 | To determine the prevalence and characteristics of minor hallucinations in AD and amnestic MCI, and to describe their potential link with cognition, behavioral symptoms, and use of psychoactive drugs. | 59.00% | NR | NR | AD | MMSE = 22.4 | NR | No acetylcholinesterase inhibitors. 30% antidepressants, 16% anxiolytics/hypnotics, 3% neuroleptics. |  | NPI-Hallucinations | MMSE |
| Sánchez-Rodríguez 2004 | Cross-sectional | 58 | To assess if the presence of depressive symptoms affects the neuropsychological performance of patients with sporadic late-onset AD. | 57.00% | NR | Late | AD | MMSE = 24.3 | NR | NR |  | Beck Depression Inventory | MMSE, Extensive cognitive testing |
| Senanarog 2005 | Cross-sectional | 73 | To study the relationship between behavioral disturbances, activities of daily living, and executive function. | 72.60% | Asian (Thai) | NR | AD | MMSE = 18.42 | NR | NR |  | NPI | Clock drawing test, Verbal fluency, MMSE |
| Serra 2010 | Longitudinal | 54 | To investigate the relationship between severity and rate of decline of the cognitive and behavioural impairment in patient with AD | 66.67% | NR | NR | AD | MMSE = 20.72 | NR | All participants on anti-cholinesterase drug |  | NPI | Mental Deterioration Battery, Phonological Word Fluency |
| Shin 2014 | Case-control | 63 | To evaluate the effects of nighttime sleep on cognition and BPSD in AD. | 73.00% | NR | NR | AD | MMSE = 16.73 | NR | No psychoactive medications. "Eight of the 64 AD patients reported that they were taking sleeping medications". |  | Pittsburgh Sleep Quality Index | Repetition, BNT, Praxis, Calculation, Shiraz Verbal Learning Test, RCFT, COWAT, Stroop |
| Soleman Hernández 2012 | Cross-sectional | 37 | To describe the presence of apathy in patients with AD. To determine whether there is a link between apathy and cognitive and motor function in these patients. To assess whether there are differences in cognitive and motor function depending on the level of apathy in patients. | 78.00% | NR | NR | AD | MMSE = 17.2 | NR | NR |  | NPI-Apathy | MMSE, Montreal Cognitive Assessment, Frontal Assessment Battery, Verbal fluency, Clock drawing test |
| Starkstein 2004 | Cross-sectional | 150 | To evaluate the psychometric characteristics of a structured interview for apathy, and to explore the overlap between apathy and depression in dementia. | 90.00% | NR | NR | AD | MMSE = 23.09 | NR | 10.67% neuroleptics, 12.67% antidepressants, 21.33% anxyolitics |  | Structured Interview for Apathy, DSM/Hamilton Depression Rating Scale | MMSE |
| Starr 2007 | Cross-sectional | 556 | To describe links between BPSD and cognitive ability in AD. To test if these relationships were stronger for absolute cognitive status or for cognition relative to premorbid mental ability. | 69.96% | NR | NR | AD | MMSE = 19.2 | NR | NR |  | NPI | MMSE, National Adult Reading Test, HVLT, Verbal fluency, Pair associates learning, Delayed matching to sample |
| Strauss 2002 | Cross-sectional | 100 | To standardize a new rating scale for the assessment of apathy in AD and report on its reliability, structure, and relation to other clinical features of AD. | 50.00% | 86% Euro-American | NR | AD | MMSE = 18.55; CDR = 1.62 | NR | NR |  | DAIR Apathy, BRSD Depression | MMSE |
| Sultzer 1992 | Cross-sectional | 61 | To study the link between cognitive dysfunction and extent of psychiatric and behavioral disturbances in dementia patients. | NR | NR | NR | AD | MMSE = 10 | NR | NR |  | Hamilton Depression Scale | MMSE |
| Sultzer 2014 | Cross-sectional | 88 | To explore the clinical associations among delusions, memory deficits, and poor insight, examine neurobiological correlates for these symptoms, and identify shared mechanisms. | 19.00% | 62.5% White; 27.3% African American; 3.4% Hispanic; 5.7% Asian/Pacific Islander. | NR | AD | MMSE = 19.3 | NR | 32 on a stable dose (3 months or more) of cholinesterase inhibitor medication and 21 on a stable dose of selective serotonin reuptake inhibitor antidepressant. No participants were being treated with an antipsychotic, benzodiazepine, or other psychotropic medication. |  | NPI- Delusions | MMSE |
| van der Mussele 2012 | Cross-sectional | 402 | To determine the prevalence of depressive symptoms in MCI and AD, and to characterise the behavior linked with significant depressive symptoms in these patients. | 67.00% | 100% Caucasian origin. | Late (76.8) | AD | MMSE = 15.2, Global Deterioration Scale = 5.1 | Yes (Inpatients and outpatients) | 16.7% no psychotropic medication, 38.8% antidepressants, 45.3% antipsychotics, 23.6% hypnotics, sedatives and anxiolytics, 36.8% cholinesterase inhibitors, 1.5% antiparkinsonian agents, 1.5% antiepileptics |  | Cornell Scale for Depression in Dementia | MMSE |
| van der Mussele 2015 | Cross-sectional | 393 | To study the prevalence of agitation in MCI and AD, and to characterise the associated behavioral symptoms. | 67.00% | 100% Caucasian origin. | Late (76.9) | AD | MMSE = 15.1 | NR | 15.6% no medication; 39.7% antidepressants; 46.3% antipsychotics; 23.4% hypnotics, sedatives, anxiolytics; 37.7% cholinesterase inhibitors; 1.5% antiparkinsonian agents; 1.5% antiepileptics. | Same cohort as above but different analysis, treated as two separate cross-sectional studies | Cohen-Mansfield Agitation Inventory | MMSE |
| Wagner 1995 | Cross-sectional | 614 | To explore the nature of behavioural problems of dementia residents and the relationship of age, gender, and the level of cognitive functioning to such problems. | 69.00% | 93% white | NR | Primarily AD | MMSE = 7.8 | No | NR |  | MBPC | MMSE |
| Welsh 1996 | Cross-sectional | 18 | To explore the association of aggressive behaviour with language impairment and other cognitive impairment. | 94.40% | NR | NR | AD | NR | No | NR |  | Yudofsky Overt Aggression | MMSE, Cambridge Cognition Examination |
| Wu 2014 | Cross-sectional | 179 | To determine the prevalence and predictors of hyperphagic behaviors in institutionalised patients with dementia. | 39.00% | NR | NR | NR | NR | Yes (Inpatients and outpatients) | NR |  | Hyperphagia questionnaire | Cognitive Abilities Screening Instrument |
| Yeager 2008 | Cross-sectional | 68 | To study the link of apathy and depression in dementia and apathy's association with basic and instrumental activities of daily living and quality of life." | 60.00% | 85% Euro-American | NR | AD, Dementia-NOS | NR | NR | NR |  | Cornell Scale for Depression in Dementia | MMSE |
| Zahodne 2015 | Longitudinal | 517 | To study cross-sectional and longitudinal relationships between individual BPSD and cognition, and between dependence and individual BPSD independent of cognitive decline. | 57.00% | 93.2% white | NR | AD | NR | NR | 31.1% antidementia medications; 21.3% antidepressant; 1.2% antipsychotic agents |  | Columbia University Scale for Psychopathology in Alzheimer’s Disease, Hamilton Depression Rating Scale | Modified MMSE |
| AD = Alzheimer's disease, BNT = Boston Naming Test, BPSD = Behavioural and psychological symptoms of dementia, BVRT = Benton Visual Retention Test, BVMT-R = Brief Visuospatial Memory test, CDR = Clinical Dementia Rating, COWAT = Controlled Oral Word Association Test, CVLT-II = California Verbal Learning Test II, Dementia-NOS = Dementia not otherwise specified, DLB = Dementia with Lewy Bodies, FTD = Frontotemporal dementia, HVLT-R = Hopkins Verbal Learning Test-Revised MCI = Mild cognitive impairment, MDRS = Mattis Dementia Rating Scale, MMSE = Mini Mental State Examination, MID = Multi-infarct dementia, NPS = Neuropsychiatric symptoms, NPI = Neuropsychiatric Inventory, NR = Not reported, PPA = Primary progressive aphasia, PDD = Parkinson's disease dementia, RCFT = Rey Complex Figure Test, SCOPA-Cog = Scales for Outcomes in PArkinson's disease-COGnition, SIVD = subcortical ischemic vascular dementia, TMT = Trail making test, UK = United Kingdom, USA = United States of America, VaD = Vascular dementia, VOSP = Visual Object Space perception Battery, WMS-R = Wechsler Memory Scale-Revised | | | | | | | | | | | | | |
