# Supplementary material for: The Associations Between Neuropsychiatric Symptoms and Cognition in People with Dementia: A Systematic Review and Meta-Analysis
Source: Neuropsychol Rev. 2023 Jul 21;34(2):581–97. doi: 10.1007/s11065-023-09608-0 (PMC11166771; doi:10.1007/s11065-023-09608-0)
Supplement: Supplementary file 3 — Supplementary file3 (DOCX 38 KB) [file 11065_2023_9608_MOESM3_ESM.docx]

**The associations between neuropsychiatric symptoms and cognition in people with dementia: A systematic review and meta-analysis**

*Neuropsychology review*

Ms. Julieta Sabates, The University of Melbourne, Australia.

Ms. Wei-Hsuan Chiu, The University of Melbourne, Australia.

A/Prof Samantha Loi, The University of Melbourne, Royal Melbourne Hospital, Australia.

Dr. Amit Lampit, The University of Melbourne, Australia.

Dr. Hanna M Gavelin, The University of Melbourne, Australia; Department of Psychology, Umea University, Sweden.

Dr. Terence Chong, The University of Melbourne, St Vincent’s Hospital Melbourne, Royal Melbourne Hospital, Australia.

Ms. Nathalie Launder, The University of Melbourne, Australia.

Dr.Anita MY Goh, National Ageing Research Institute; The University of Melbourne, Australia.

Prof. Amy Brodtmann, Cognitive Health Initiative, Central Clinical School, Monash University, Australia.

Prof. Nicola Lautenschlager, The University of Melbourne, Australia.

A/Prof. Alex Bahar-Fuchs, The University of Melbourne, Australia.

Corresponding author: Ms Julieta Sabates. Mailing address: 151 Barry Street, Carlton 3053, Victoria, Australia; Email address: Julieta.sabates@unimelb.edu.au

Supplementary material: S3- Study quality assessment tables

Quality assessment of cross-sectional and longitudinal studies

| tudy | Design | Was the research question or objective in this paper clearly stated? | Was the study population clearly specified and defined? | Were all the subjects selected or recruited from the same or similar populations (including the same time period)? Were inclusion and exclusion criteria for being in the study prespecified and applied uniformly to all participants? | For exposures that can vary in amount or level, did the study examine different levels of the exposure as related to the outcome (e.g., categories of exposure, or exposure measured as continuous variable)? | Were the exposure measures (independent variables) clearly defined, valid, reliable, and implemented consistently across all study participants? | Were the outcome measures (dependent variables) clearly defined, valid, reliable, and implemented consistently across all study participants? | Were the outcome assessors blinded to the exposure status of participants? | Were key potential confounding variables measured and adjusted statistically for their impact on the relationship between exposure(s) and outcome(s)? |
| --- | --- | --- | --- | --- | --- | --- | --- | --- | --- |
| Akyol 2020 | Cross-sectional | Yes | Yes | Yes | Yes | Yes | Yes | NR | Yes |
| Balci 2011 | Cross-sectional | Yes | Yes | Yes | Yes | Yes | Yes | NR | Yes |
| Bhat 2021 | Cross-sectional | Yes | Yes | Yes | Yes | Yes | Yes | NR | No |
| Breitve 2018 | Longitudinal | Yes | Yes | Yes | Yes | Yes | Yes | NR | Yes |
| Bronnick 2011 | Cross-sectional | Yes | Yes | Yes | No | Yes | Yes | Yes | Yes |
| Bylsma 1994 | Longitudinal | Yes | No | Yes | No | Yes | Yes | NR | No |
| Camargo 2017 | Cross-sectional | No | No | NR | Yes | Yes | Yes | NR | No |
| Chwiszczuk 2017 | Longitudinal | Yes | Yes | NR | NA | Yes | Yes | NR | Yes |
| D'antonio 2019 | Longitudinal | Yes | No | Yes | Yes | Yes | Yes | NR | Yes |
| de Oliveira 2015 - "Correlations" | Cross-sectional | Yes | Yes | Yes | Yes | Yes | Yes | NR | Yes |
| de Oliveira 2015 2 | Cross-sectional | Yes | Yes | Yes | Yes | Yes | Yes | NR | No |
| de Oliveira 2020 | Cross-sectional | Yes | Yes | Yes | Yes | Yes | Yes | NR | No |
| Demichele-Sweet 2011 | Longitudinal | Yes | Yes | Yes | Yes | Yes | Yes | NR | Yes |
| de Paula 2016 | Cross-sectional | Yes | No | Yes | Yes | Yes | Yes | NR | Yes |
| Drijgers 2011 | Cross-sectional | Yes | No | Yes | Yes | Yes | Yes | NR | Yes |
| Eikelboom 2021 | Longitudinal | Yes | Yes | Yes | Yes | Yes | Yes | NR | Yes |
| Eustace 2001 | Cross-sectional | Yes | No | Yes | Yes | Yes | Yes | NR | Yes |
| Fahlander 1999 | Longitudinal | Yes | Yes | Yes | No | Yes | Yes | NR | No |
| Fernandez 2010 | Cross-sectional | Yes | Yes | Yes | Yes | Yes | Yes | NR | No |
| Fernandez Martinez 2010a | Cross-sectional | Yes | Yes | Yes | Yes | Yes | Yes | Yes | No |
| Fillit 2021 | Cross-sectional | Yes | Yes | Yes | Yes | Yes | Yes | NR | Yes |
| Fitz 1994 | Cross-sectional | Yes | Yes | Yes | Yes | Yes | Yes | Yes | Yes |
| Flynn 1991a | Cross-sectional | No | No | No | Yes | Yes | Yes | No | No |
| Gallo 2008 | Cross-sectional | Yes | No | Yes | Yes | Yes | Yes | NR | No |
| Galynker 1995 | Cross-sectional | Yes | No | Yes | Yes | Yes | Yes | NR | No |
| Gilley 1991 | Cross-sectional | Yes | Yes | Yes | Yes | Yes | Yes | NR | Yes |
| Grossi 2013 | Cross-sectional | Yes | No | No | Yes | Yes | Yes | NR | No |
| Hallikainen 2012 | Longitudinal | Yes | Yes | Yes | Yes | Yes | Yes | NR | No |
| Harwood 2000 | Cross-sectional | Yes | No | Yes | Yes | Yes | Yes | NR | Yes |
| Hopkins 2005 | Cross-sectional | Yes | No | NR | Yes | Yes | Yes | No | No |
| Ito 2007 | Longitudinal | Yes | Yes | NR | Yes | Yes | Yes | Yes | No |
| Janzing 2005 | Cross-sectional | Yes | No | Yes | Yes | Yes | Yes | NR | No |
| Keator 2019a | Cross-sectional | Yes | No | No | Yes | Yes | Yes | No | No |
| Kuzis 1999 | Cross-sectional | Yes | Yes | Yes | Yes | Yes | Yes | Yes | No |
| Kwak 2013 | Cross-sectional | Yes | No | NR | Yes | Yes | Yes | NR | No |
| Lam 2006 | Cross-sectional | Yes | Yes | NR | Yes | Yes | NR | No | No |
| Lee 2012 | Cross-sectional | Yes | Yes | NR | Yes | Yes | Yes | NR | No |
| Lee 2019 | Cross-sectional | Yes | Yes | NR | Yes | Yes | Yes | NR | Yes |
| Levy 1998a | Cross-sectional | Yes | Yes | NR | Yes | Yes | Yes | NR | No |
| Logsdon 1998 | Longitudinal | Yes | Yes | NR | Yes | Yes | Yes | NR | No |
| Lopez 1991 | Longitudinal | Yes | Yes | Yes | Yes | Yes | Yes | Yes | Yes |
| Machado 2020 | Cross-sectional | Yes | Yes | Yes | Yes | Yes | Yes | NR | Yes |
| Mc Pherson 2002 | Cross-sectional | Yes | No | Yes | Yes | Yes | Yes | NR | Yes |
| Migliorelli 1995 | Cross-sectional | Yes | Yes | Yes | No | Yes | Yes | No | Yes |
| Mizrahi 2006 | Cross-sectional | Yes | Yes | Yes | Yes | Yes | Yes | NR | Yes |
| Montagnese 2021 | Cross-sectional | Yes | No | No | Yes | Yes | Yes | NR | No |
| Na 2017 | Cross-sectional | Yes | No | No | Yes | Yes | Yes | NR | Yes |
| Nagata 2010 | Cross-sectional | Yes | No | Yes | No | Yes | Yes | No | Yes |
| Nagata 2017 | Cross-sectional | Yes | Yes | Yes | Yes | Yes | Yes | No | Yes |
| Nakaaki 2007 | Cross-sectional | Yes | Yes | Yes | No | Yes | Yes | Yes | Yes |
| Nakaaki 2008 | Cross-sectional | Yes | Yes | Yes | No | Yes | Yes | Yes | NR |
| Nakatsuka 2014 | Cross-sectional | Yes | No | Yes | Yes | Yes | Yes | Yes | Yes |
| Onyike 2007 | Longitudinal | Yes | Yes | Yes | Yes | Yes | Yes | NR | Yes |
| Pagonabarraga 2008 | Cross-sectional | Yes | Yes | Yes | No | Yes | Yes | NR | No |
| Park 2019 | Cross-sectional | Yes | Yes | Yes | Yes | Yes | Yes | NR | Yes |
| Perri 2014 | Cross-sectional | Yes | No | Yes | Yes | Yes | Yes | No | Yes |
| Perri 2018 | Cross-sectional | Yes | Yes | No | Yes | Yes | Yes | NR | No |
| Qian 2018 | Longitudinal | Yes | Yes | Yes | No | Yes | Yes | NR | Yes |
| Quaranta 2015 | Cross-sectional | Yes | Yes | Yes | No | Yes | Yes | NR | Yes |
| Reed 1993 | Cross-sectional | Yes | No | NR | No | Yes | Yes | NR | Yes |
| Rolland 2007 | Longitudinal | Yes | Yes | Yes | No | Yes | Yes | NR | Yes |
| Ross 1998 | Cross-sectional | Yes | Yes | Yes | Yes | Yes | Yes | NR | Yes |
| Rozum 2019 | Cross-sectional | Yes | Yes | Yes | Yes | Yes | Yes | NR | Yes |
| Ruiz 2018 | Cross-sectional | Yes | Yes | Yes | Yes | Yes | Yes | No | Yes |
| Sánchez-Rodríguez 2004 | Cross-sectional | Yes | Yes | NR | Yes | Yes | Yes | NR | No |
| Senanarog 2005a | Cross-sectional | Yes | Yes | Yes | Yes | Yes | Yes | NR | Yes |
| Serra 2010 | Longitudinal | Yes | Yes | Yes | Yes | Yes | Yes | NR | No |
| Soleman Hernández 2012 | Cross-sectional | Yes | Yes | Yes | Yes | Yes | Yes | Yes | No |
| Starkstein 2004 | Cross-sectional | Yes | Yes | Yes | Yes | Yes | Yes | Yes | Yes |
| Starr 2007 | Cross-sectional | Yes | Yes | Yes | Yes | Yes | Yes | No | No |
| Strauss 2002 | Cross-sectional | Yes | No | Yes | Yes | Yes | Yes | NR | No |
| Sultzer 1992 | Cross-sectional | Yes | No | Yes | Yes | Yes | Yes | NR | No |
| Sultzer 2014 | Cross-sectional | Yes | Yes | Yes | Yes | Yes | Yes | No | No |
| van der Mussele 2012 | Cross-sectional | Yes | Yes | Yes | Yes | Yes | Yes | No | No |
| van der Mussele 2015 | Cross-sectional | Yes | Yes | Yes | Yes | Yes | Yes | No | Yes |
| Wagner 1995 | Cross-sectional | Yes | Yes | Yes | Yes | Yes | No | NR | No |
| Welsh 1996 | Cross-sectional | Yes | Yes | Yes | Yes | Yes | Yes | No | No |
| Wu 2014 | Cross-sectional | Yes | Yes | Yes | Yes | Yes | Yes | No | Yes |
| Yeager 2008 | Cross-sectional | Yes | Yes | Yes | Yes | Yes | Yes | NR | No |
| Zahodne 2015 | Longitudinal | Yes | Yes | Yes | Yes | Yes | Yes | NR | Yes |

Quality assessment of cross-sectional studies

| Was the study population clearly specified and defined? | Did the authors include a sample size justification? | Were controls selected or recruited from the same or similar population that gave rise to the cases (including the same timeframe)? | Were the definitions, inclusion and exclusion criteria, algorithms or processes used to identify or select cases and controls valid, reliable, and implemented consistently across all study participants? | Were the cases clearly defined and differentiated from controls? | If less than 100 percent of eligible cases and/or controls were selected for the study, were the cases and/or controls randomly selected from those eligible? | Was there use of concurrent controls? | Were the investigators able to confirm that the exposure/risk occurred prior to the development of the condition or event that defined a participant as a case? | Were the measures of exposure/risk clearly defined, valid, reliable, and implemented consistently (including the same time period) across all study participants? | Were the assessors of exposure/risk blinded to the case or control status of participants? | Were key potential confounding variables measured and adjusted statistically in the analyses? If matching was used, did the investigators account for matching during study analysis? |
| --- | --- | --- | --- | --- | --- | --- | --- | --- | --- | --- |
| No | No | NR | No | Yes | NR | NR | Yes | Yes | NR | No |
| Yes | No | NR | Yes | Yes | NR | NR | NR | Yes | NR | Yes |
| No | No | Yes | Yes | Yes | NA | NR | NR | Yes | NR | No |
| Yes | No | No | No | No | No | NR | Yes | Yes | NR | No |
| Yes | No | NR | Yes | Yes | NR | NR | NR | Yes | NR | No |
| Yes | Yes | No | Yes | Yes | NR | NR | Yes | Yes | NR | No |
| Yes | No | No | Yes | No | No | NR | Yes | Yes | NR | Yes |
| Yes | No | No | Yes | Yes | NR | NR | Yes | Yes | NR | No |
| No | No | No | NR | Yes | NR | NR | No | Yes | No | No |
| Yes | Yes | Yes | Yes | Yes | NA | NR | NR | Yes | No | Yes |
