# Supplementary material for: The Associations Between Neuropsychiatric Symptoms and Cognition in People with Dementia: A Systematic Review and Meta-Analysis
Source: Neuropsychol Rev. 2023 Jul 21;34(2):581–97. doi: 10.1007/s11065-023-09608-0 (PMC11166771; doi:10.1007/s11065-023-09608-0)
Supplement: Supplementary file 4 — Supplementary file4 (DOCX 93 KB) [file 11065_2023_9608_MOESM4_ESM.docx]

**The associations between neuropsychiatric symptoms and cognition in people with dementia: A systematic review and meta-analysis**

*Neuropsychology review*

Ms. Julieta Sabates, The University of Melbourne, Australia.

Ms. Wei-Hsuan Chiu, The University of Melbourne, Australia.

A/Prof Samantha Loi, The University of Melbourne, Royal Melbourne Hospital, Australia.

Dr. Amit Lampit, The University of Melbourne, Australia.

Dr. Hanna M Gavelin, The University of Melbourne, Australia; Department of Psychology, Umea University, Sweden.

Dr. Terence Chong, The University of Melbourne, St Vincent’s Hospital Melbourne, Royal Melbourne Hospital, Australia.

Ms. Nathalie Launder, The University of Melbourne, Australia.

Dr.Anita MY Goh, National Ageing Research Institute; The University of Melbourne, Australia.

Prof. Amy Brodtmann, Cognitive Health Initiative, Central Clinical School, Monash University, Australia.

Prof. Nicola Lautenschlager, The University of Melbourne, Australia.

A/Prof. Alex Bahar-Fuchs, The University of Melbourne, Australia.

Corresponding author: Ms Julieta Sabates. Mailing address: 151 Barry Street, Carlton 3053, Victoria, Australia; Email address: Julieta.sabates@unimelb.edu.au

Supplementary material: S4- Table of all results

| Diagnosis | NPS | Cognition | K | Estimate | Std Error | I. sq. | Tau sq. | dfs | P(t) | 95%CI L. | 95% CI U. |
| --- | --- | --- | --- | --- | --- | --- | --- | --- | --- | --- | --- |
| All | Affective | Attention | 14.00 | -0.15 | 0.05 | 39.53 | 0.04 | 9.78 | 0.011 | -0.26 | -0.04 |
| All | Affective | Executive function | 31.00 | -0.03 | 0.10 | 80.19 | 0.18 | 28.50 | 0.803 | -0.24 | 0.19 |
| All | Affective | Global cognition | 46.00 | -0.09 | 0.04 | 49.86 | 0.04 | 33.90 | 0.028 | -0.16 | -0.01 |
| All | Affective | Memory | 23.00 | -0.01 | 0.08 | 64.73 | 0.07 | 18.80 | 0.884 | -0.19 | 0.16 |
| All | Affective | Semantic knowledge | 20.00 | -0.15 | 0.05 | 28.84 | 0.01 | 10.50 | 0.007 | -0.25 | -0.05 |
| All | Affective | Social cognition | 2.00 | 0.02 | 0.37 | 52.75 | 0.25 | 1.00 | 0.962 | -4.64 | 4.68 |
| All | Affective | Speed | 13.00 | -0.16 | 0.08 | 45.24 | 0.05 | 10.10 | 0.066 | -0.34 | 0.01 |
| All | Affective | Visuospatial skills | 18.00 | -0.09 | 0.15 | 80.76 | 0.20 | 16.20 | 0.55 | -0.41 | 0.23 |
| All | Affective | Working memory | 16.00 | -0.05 | 0.04 | 0.00 | 0.00 | 5.62 | 0.26 | -0.16 | 0.05 |
| All | Aggression | Attention | 4.00 | -0.37 | 0.33 | 74.26 | 0.33 | 2.88 | 0.347 | -1.45 | 0.70 |
| All | Aggression | Executive function | 7.00 | -0.19 | 0.15 | 56.88 | 0.08 | 5.23 | 0.247 | -0.57 | 0.18 |
| All | Aggression | Global cognition | 16.00 | -0.21 | 0.05 | 67.64 | 0.03 | 12.10 | 0.001 | -0.33 | -0.10 |
| All | Aggression | Memory | 3.00 | -0.50 | 0.30 | 73.74 | 0.21 | 1.78 | 0.251 | -1.95 | 0.95 |
| All | Aggression | Semantic knowledge | 3.00 | -0.65 | 0.46 | 86.56 | 0.49 | 1.87 | 0.304 | -2.77 | 1.48 |
| All | Aggression | Visuospatial skills | 2.00 | -0.63 | 0.44 | 71.47 | 0.50 | 1.00 | 0.39 | -6.24 | 4.98 |
| All | Aggression | Working memory | 5.00 | -0.18 | 0.09 | 29.39 | 0.03 | 2.63 | 0.164 | -0.49 | 0.14 |
| All | Circadian rhythms | Attention | 5.00 | -0.89 | 0.77 | 92.04 | 0.77 | 3.94 | 0.264 | -2.79 | 1.02 |
| All | Circadian rhythms | Executive function | 5.00 | -0.21 | 0.20 | 71.77 | 0.16 | 3.88 | 0.364 | -0.77 | 0.36 |
| All | Circadian rhythms | Global cognition | 9.00 | -0.26 | 0.16 | 82.29 | 0.11 | 7.54 | 0.15 | -0.63 | 0.12 |
| All | Circadian rhythms | Memory | 3.00 | -0.08 | 0.18 | 57.45 | 0.07 | 1.92 | 0.686 | -0.89 | 0.72 |
| All | Circadian rhythms | Semantic knowledge | 3.00 | -0.16 | 0.15 | 60.25 | 0.08 | 1.93 | 0.416 | -0.84 | 0.54 |
| All | Circadian rhythms | Visuospatial skills | 4.00 | -0.24 | 0.14 | 35.38 | 0.03 | 2.51 | 0.2 | -0.73 | 0.26 |
| All | Circadian rhythms | Working memory | 4.00 | -0.15 | 0.07 | 0.00 | 0.00 | 2.22 | 0.155 | -0.43 | 0.13 |
| All | Dysexecutive | Attention | 3.00 | 0.18 | 0.14 | 0.00 | 0.00 | 1.94 | 0.327 | -0.42 | 0.78 |
| All | Dysexecutive | Executive function | 7.00 | -0.03 | 0.11 | 42.63 | 0.04 | 4.74 | 0.782 | -0.31 | 0.25 |
| All | Dysexecutive | Global cognition | 10.00 | -0.22 | 0.12 | 64.69 | 0.07 | 8.00 | 0.094 | -0.50 | 0.05 |
| All | Dysexecutive | Memory | 2.00 | -0.04 | 0.02 | 0.00 | 0.00 | 1.00 | 0.25 | -0.26 | 0.18 |
| All | Dysexecutive | Semantic knowledge | 2.00 | -0.05 | 0.01 | 0.00 | 0.00 | 1.00 | 0.145 | -0.18 | 0.09 |
| All | Dysexecutive | Working memory | 5.00 | -0.06 | 0.05 | 0.00 | 0.00 | 1.72 | 0.396 | -0.33 | 0.21 |
| All | Motor disturbances | Attention | 4.00 | -1.26 | 1.04 | 93.80 | 1.68 | 2.98 | 0.316 | -4.59 | 2.08 |
| All | Motor disturbances | Executive function | 6.00 | -0.13 | 0.08 | 15.80 | 0.01 | 2.65 | 0.223 | -0.42 | 0.15 |
| All | Motor disturbances | Global cognition | 13.00 | -0.46 | 0.13 | 83.09 | 0.12 | 11.10 | 0.004 | -0.74 | -0.18 |
| All | Motor disturbances | Memory | 2.00 | -0.27 | 0.07 | 0.00 | 0.00 | 1.00 | 0.158 | -1.12 | 0.59 |
| All | Motor disturbances | Semantic knowledge | 2.00 | -0.27 | 0.03 | 0.00 | 0.00 | 1.00 | 0.0654 | -0.63 | 0.08 |
| All | Motor disturbances | Working memory | 4.00 | -0.22 | 0.07 | 0.00 | 0.00 | 1.53 | 0.13 | -0.63 | 0.20 |
| All | Overall NPS | Attention | 6.00 | -0.18 | 0.01 | 0.00 | 0.00 | 1.35 | 0.013 | -0.25 | -0.12 |
| All | Overall NPS | Executive function | 12.00 | -0.31 | 0.06 | 46.96 | 0.02 | 6.36 | 0.001 | -0.45 | -0.17 |
| All | Overall NPS | Global cognition | 22.00 | -0.36 | 0.04 | 0.36 | 0.07 | 16.70 | 0.00005 | -0.51 | -0.22 |
| All | Overall NPS | Memory | 5.00 | -0.21 | 0.03 | 0.00 | 0.00 | 1.96 | 0.016 | -0.32 | -0.10 |
| All | Overall NPS | Semantic knowledge | 8.00 | -0.19 | 0.04 | 35.87 | 0.01 | 2.82 | 0.027 | -0.33 | -0.04 |
| All | Overall NPS | Visuospatial skills | 4.00 | -0.10 | 0.08 | 65.14 | 0.02 | 1.83 | 0.373 | -0.48 | 0.29 |
| All | Overall NPS | Working memory | 5.00 | -0.21 | 0.12 | 8.88 | 0.01 | 2.08 | 0.214 | -0.71 | 0.29 |
| All | Psychosis | Attention | 11.00 | -0.30 | 0.21 | 84.84 | 0.37 | 9.75 | 0.187 | -0.78 | 0.17 |
| All | Psychosis | Executive function | 16.00 | -0.17 | 0.08 | 54.52 | 0.06 | 13.10 | 0.0437 | -0.33 | -0.01 |
| All | Psychosis | Global cognition | 36.00 | -0.40 | 0.07 | 81.86 | 0.09 | 32.10 | 1.19E-06 | -0.54 | -0.27 |
| All | Psychosis | Memory | 14.00 | -0.14 | 0.08 | 62.56 | 0.08 | 11.70 | 0.096 | -0.30 | 0.03 |
| All | Psychosis | Semantic knowledge | 14.00 | -0.15 | 0.09 | 60.87 | 0.07 | 11.50 | 0.13 | -0.35 | 0.05 |
| All | Psychosis | Speed | 4.00 | 0.29 | 0.11 | 53.10 | 0.10 | 2.67 | 0.0961 | -0.10 | 0.68 |
| All | Psychosis | Visuospatial skills | 13.00 | -0.10 | 0.09 | 34.29 | 0.03 | 10.10 | 0.268 | -0.30 | 0.09 |
| All | Psychosis | Working memory | 10.00 | -0.22 | 0.06 | 4.11 | 0.00 | 4.01 | 0.022 | -0.38 | -0.05 |
| All | Aggression | Attention | 3.00 | -1.80 | 1.70 | 96.91 | 8.53 | 2.00 | 0.401 | -9.11 | 5.51 |
| All | Aggression | Executive function | 4.00 | 0.04 | 0.04 | 0.00 | 0.00 | 1.24 | 0.51 | -0.29 | 0.36 |
| All | Aggression | Global cognition | 9.00 | -0.43 | 0.23 | 82.91 | 0.18 | 7.33 | 0.103 | -0.97 | 0.11 |
| All | Aggression | Memory | 2.00 | -0.47 | 0.54 | 77.81 | 0.57 | 1.00 | 0.541 | -7.26 | 6.32 |
| All | Aggression | Semantic knowledge | 2.00 | -0.71 | 0.92 | 89.06 | 1.73 | 1.00 | 0.582 | -12.40 | 11.00 |
| All | Aggression | Working memory | 3.00 | -0.14 | 0.14 | 37.99 | 0.05 | 1.46 | 0.455 | -1.00 | 0.73 |
| All | Agitation | Executive function | 2.00 | -0.38 | 0.32 | 83.27 | 0.18 | 1.00 | 0.445 | -4.47 | 3.71 |
| All | Agitation | Global cognition | 8.00 | -0.24 | 0.08 | 78.00 | 0.04 | 6.47 | 0.023 | -0.43 | -0.04 |
| All | Anxiety | Attention | 3.00 | -0.53 | 0.56 | 85.78 | 0.67 | 1.99 | 0.445 | -2.95 | 1.89 |
| All | Anxiety | Executive function | 4.00 | -0.11 | 0.06 | 0.00 | 0.00 | 1.34 | 0.235 | -0.51 | 0.28 |
| All | Anxiety | Global cognition | 10.00 | 0.00 | 0.06 | 16.91 | 0.01 | 5.99 | 0.995 | -0.15 | 0.15 |
| All | Anxiety | Working memory | 3.00 | 0.33 | 0.28 | 78.13 | 0.20 | 1.94 | 0.359 | -0.90 | 1.56 |
| All | Apathy | Attention | 6.00 | -0.10 | 0.06 | 0.00 | 0.00 | 4.60 | 0.132 | -0.25 | 0.05 |
| All | Apathy | Executive function | 19.00 | -0.13 | 0.08 | 59.69 | 0.08 | 15.90 | 0.099 | -0.29 | 0.03 |
| All | Apathy | Global cognition | 24.00 | -0.28 | 0.06 | 37.09 | 0.03 | 17.10 | 0.002 | -0.41 | -0.16 |
| All | Apathy | Memory | 12.00 | -0.16 | 0.04 | 0.00 | 0.00 | 4.46 | 0.009 | -0.26 | -0.06 |
| All | Apathy | Semantic knowledge | 12.00 | -0.19 | 0.05 | 7.06 | 0.00 | 4.96 | 0.014 | -0.31 | -0.06 |
| All | Apathy | Social cognition | 2.00 | 0.02 | 0.37 | 52.75 | 0.25 | 1.00 | 0.962 | -4.64 | 4.68 |
| All | Apathy | Speed | 8.00 | -0.07 | 0.05 | 5.13 | 0.00 | 4.28 | 0.26 | -0.21 | 0.07 |
| All | Apathy | Visuospatial skills | 8.00 | -0.26 | 0.08 | 0.00 | 0.00 | 4.79 | 0.026 | -0.47 | -0.05 |
| All | Apathy | Working memory | 12.00 | -0.18 | 0.06 | 0.00 | 0.00 | 4.01 | 0.044 | -0.35 | -0.01 |
| All | Delusions | Attention | 7.00 | -0.08 | 0.14 | 23.76 | 0.02 | 4.91 | 0.598 | -0.43 | 0.28 |
| All | Delusions | Executive function | 10.00 | -0.32 | 0.11 | 46.95 | 0.04 | 7.21 | 0.019 | -0.58 | -0.07 |
| All | Delusions | Global cognition | 22.00 | -0.31 | 0.06 | 65.99 | 0.05 | 18.20 | 0.00009 | -0.44 | -0.18 |
| All | Delusions | Memory | 8.00 | -0.17 | 0.09 | 45.67 | 0.04 | 5.45 | 0.1 | -0.39 | 0.05 |
| All | Delusions | Semantic knowledge | 9.00 | -0.24 | 0.09 | 45.66 | 0.03 | 6.18 | 0.037 | -0.45 | -0.02 |
| All | Delusions | Visuospatial skills | 7.00 | -0.01 | 0.06 | 0.00 | 0.00 | 4.17 | 0.913 | -0.17 | 0.16 |
| All | Delusions | Working memory | 5.00 | -0.30 | 0.09 | 0.00 | 0.00 | 1.66 | 0.0965 | -0.76 | 0.59 |
| All | Depression | Attention | 6.00 | -0.23 | 0.07 | 7.12 | 0.00 | 1.68 | 0.1 | -0.59 | 0.13 |
| All | Depression | Executive function | 11.00 | 0.15 | 0.25 | 89.99 | 0.28 | 9.56 | 0.557 | -0.41 | 0.71 |
| All | Depression | Global cognition | 27.00 | 0.03 | 0.05 | 39.45 | 0.02 | 16.10 | 0.529 | -0.07 | 0.13 |
| All | Depression | Memory | 11.00 | 0.16 | 0.16 | 78.65 | 0.12 | 8.96 | 0.337 | -0.20 | 0.52 |
| All | Depression | Semantic knowledge | 8.00 | -0.13 | 0.11 | 59.69 | 0.04 | 4.93 | 0.3 | -0.42 | 0.16 |
| All | Depression | Speed | 4.00 | -0.28 | 0.30 | 67.87 | 0.22 | 2.90 | 0.423 | -1.23 | 0.68 |
| All | Depression | Visuospatial skills | 9.00 | 0.10 | 0.29 | 89.64 | 0.43 | 7.79 | 0.747 | -0.58 | 0.78 |
| All | Depression | Working memory | 4.00 | 0.05 | 0.05 | 11.62 | 0.01 | 1.65 | 0.462 | -0.24 | 0.34 |
| All | Disinhibition | Attention | 3.00 | 0.18 | 0.14 | 0.00 | 0.00 | 1.94 | 0.327 | -0.42 | 0.78 |
| All | Disinhibition | Executive function | 6.00 | -0.02 | 0.12 | 42.78 | 0.04 | 3.96 | 0.881 | -0.34 | 0.31 |
| All | Disinhibition | Global cognition | 9.00 | -0.23 | 0.13 | 69.00 | 0.08 | 7.31 | 0.121 | -0.53 | 0.08 |
| All | Disinhibition | Memory | 2.00 | -0.01 | 0.03 | 0.00 | 0.00 | 1.00 | 0.782 | -0.37 | 0.35 |
| All | Disinhibition | Semantic knowledge | 2.00 | -0.02 | 0.00 | 0.00 | 0.00 | 1.00 | 0.0602 | -0.04 | 0.00 |
| All | Disinhibition | Working memory | 4.00 | -0.01 | 0.05 | 0.00 | 0.00 | 1.52 | 0.81 | -0.32 | 0.29 |
| All | Dysphoria | Attention | 2.00 | -0.10 | 0.00 | 0.00 | 0.00 | 1.00 | 0.004 | -0.11 | -0.09 |
| All | Dysphoria | Executive function | 3.00 | -0.10 | 0.26 | 50.93 | 0.10 | 1.99 | 0.734 | -1.20 | 1.00 |
| All | Dysphoria | Global cognition | 4.00 | -0.15 | 0.07 | 0.00 | 0.00 | 1.80 | 0.16 | -0.47 | 0.17 |
| All | Dysphoria | Working memory | 2.00 | 0.17 | 0.07 | 0.00 | 0.00 | 1.00 | 0.259 | -0.74 | 1.07 |
| All | Eating disturbances | Attention | 3.00 | -0.28 | 0.13 | 0.00 | 0.00 | 1.56 | 0.194 | -1.02 | 0.45 |
| All | Eating disturbances | Executive function | 3.00 | -0.27 | 0.30 | 76.96 | 0.18 | 1.97 | 0.464 | -1.59 | 1.05 |
| All | Eating disturbances | Global cognition | 5.00 | -0.15 | 0.05 | 0.00 | 0.00 | 1.80 | 0.117 | -0.41 | 0.10 |
| All | Eating disturbances | Memory | 2.00 | -0.14 | 0.23 | 60.04 | 0.08 | 1.00 | 0.647 | -3.03 | 2.75 |
| All | Eating disturbances | Semantic knowledge | 2.00 | -0.26 | 0.14 | 36.25 | 0.03 | 1.00 | 0.305 | -2.00 | 1.48 |
| All | Eating disturbances | Visuospatial skills | 2.00 | -0.04 | 0.00 | 0.00 | 0.00 | 1.00 | 0.044 | -0.07 | 0.00 |
| All | Eating disturbances | Working memory | 3.00 | -0.15 | 0.09 | 0.00 | 0.00 | 1.55 | 0.252 | -0.64 | 0.34 |
| All | Euphoria | Attention | 2.00 | 0.23 | 0.22 | 6.59 | 0.01 | 1.00 | 0.493 | -2.57 | 3.02 |
| All | Euphoria | Executive function | 3.00 | -0.11 | 0.16 | 0.00 | 0.00 | 1.97 | 0.55 | -0.81 | 0.58 |
| All | Euphoria | Global cognition | 6.00 | -0.04 | 0.05 | 0.00 | 0.00 | 2.95 | 0.491 | -0.21 | 0.13 |
| All | Euphoria | Working memory | 2.00 | 0.24 | 0.14 | 0.00 | 0.00 | 1.00 | 0.34 | -1.54 | 2.02 |
| All | Hallucinations | Attention | 6.00 | -0.75 | 0.53 | 90.51 | 0.76 | 4.92 | 0.217 | -2.13 | 0.62 |
| All | Hallucinations | Executive function | 7.00 | -0.11 | 0.09 | 55.58 | 0.05 | 4.96 | 0.268 | -0.33 | 0.11 |
| All | Hallucinations | Global cognition | 20.00 | -0.44 | 0.12 | 81.32 | 0.14 | 17.80 | 0.001 | -0.69 | -0.19 |
| All | Hallucinations | Memory | 4.00 | -0.14 | 0.15 | 75.75 | 0.10 | 2.71 | 0.451 | -0.65 | 0.38 |
| All | Hallucinations | Semantic knowledge | 3.00 | -0.05 | 0.22 | 79.41 | 0.09 | 1.93 | 0.828 | -1.01 | 0.91 |
| All | Hallucinations | Speed | 2.00 | 0.48 | 0.21 | 79.73 | 0.48 | 1.00 | 0.241 | -2.16 | 3.11 |
| All | Hallucinations | Visuospatial skills | 4.00 | -0.33 | 0.21 | 73.40 | 0.15 | 2.87 | 0.223 | -1.03 | 0.37 |
| All | Hallucinations | Working memory | 6.00 | -0.22 | 0.08 | 0.00 | 0.00 | 2.26 | 0.104 | -0.55 | 0.10 |
| All | Irritability | Attention | 2.00 | -0.16 | 0.10 | 0.00 | 0.00 | 1.00 | 0.368 | -1.46 | 1.15 |
| All | Irritability | Executive function | 3.00 | 0.12 | 0.14 | 0.00 | 0.00 | 1.96 | 0.479 | -0.50 | 0.75 |
| All | Irritability | Global cognition | 5.00 | -0.02 | 0.08 | 0.00 | 0.00 | 2.45 | 0.792 | -0.33 | 0.28 |
| All | Irritability | Working memory | 2.00 | 0.00 | 0.28 | 41.35 | 0.06 | 1.00 | 0.994 | -3.53 | 3.53 |
| All | Sleep disturbances | Attention | 3.00 | -0.18 | 0.17 | 22.07 | 0.02 | 1.99 | 0.406 | -0.92 | 0.56 |
| All | Sleep disturbances | Executive function | 3.00 | 0.07 | 0.19 | 28.94 | 0.03 | 1.99 | 0.752 | -0.74 | 0.87 |
| All | Sleep disturbances | Global cognition | 4.00 | -0.09 | 0.13 | 43.64 | 0.03 | 2.70 | 0.517 | -0.52 | 0.34 |
| All | Sleep disturbances | Memory | 2.00 | 0.16 | 0.24 | 46.06 | 0.06 | 1.00 | 0.625 | -2.90 | 3.23 |
| All | Sleep disturbances | Semantic knowledge | 2.00 | -0.05 | 0.19 | 58.53 | 0.10 | 1.00 | 0.828 | -2.52 | 2.41 |
| All | Sleep disturbances | Visuospatial skills | 2.00 | -0.33 | 0.05 | 0.00 | 0.00 | 1.00 | 0.098 | -0.98 | 0.32 |
| All | Sleep disturbances | Working memory | 3.00 | -0.08 | 0.16 | 15.09 | 0.01 | 1.98 | 0.666 | -0.77 | 0.61 |
| AD | Affective | Attention | 12.00 | -0.15 | 0.05 | 46.69 | 0.05 | 8.71 | 0.027 | -0.27 | -0.02 |
| AD | Affective | Executive function | 22.00 | 0.00 | 0.13 | 83.61 | 0.20 | 20.00 | 0.972 | -0.28 | 0.28 |
| AD | Affective | Global cognition | 35.00 | -0.06 | 0.04 | 50.80 | 0.03 | 24.90 | 0.145 | -0.15 | 0.02 |
| AD | Affective | Memory | 16.00 | 0.07 | 0.11 | 71.44 | 0.08 | 13.00 | 0.53 | -0.17 | 0.32 |
| AD | Affective | Semantic knowledge | 13.00 | -0.11 | 0.05 | 26.82 | 0.01 | 5.63 | 0.0698 | -0.23 | 0.01 |
| AD | Affective | Social cognition | 2.00 | 0.02 | 0.37 | 52.75 | 0.25 | 1.00 | 0.962 | -4.64 | 4.68 |
| AD | Affective | Speed | 10.00 | -0.23 | 0.11 | 55.86 | 0.09 | 7.92 | 0.0819 | -0.49 | 0.04 |
| AD | Affective | Visuospatial skills | 12.00 | 0.03 | 0.22 | 86.20 | 0.31 | 107.00 | 0.892 | -0.46 | 0.52 |
| AD | Affective | Working memory | 12.00 | -0.05 | 0.05 | 0.00 | 0.00 | 3.53 | 0.37 | -0.21 | 0.10 |
| AD | Aggression | Attention | 2.00 | -0.46 | 0.69 | 89.17 | 1.99 | 1.00 | 0.709 | -12.40 | 11.50 |
| AD | Aggression | Executive function | 5.00 | -0.12 | 0.19 | 60.28 | 0.09 | 3.43 | 0.552 | -0.68 | 0.43 |
| AD | Aggression | Global cognition | 13.00 | -0.24 | 0.06 | 42.01 | 0.02 | 9.19 | 0.000566 | -0.34 | -0.13 |
| AD | Aggression | Memory | 2.00 | -0.49 | 0.50 | 74.39 | 0.50 | 1.00 | 0.506 | -6.88 | 5.89 |
| AD | Aggression | Semantic knowledge | 2.00 | -0.76 | 0.88 | 88.34 | 1.77 | 1.00 | 0.545 | -11.90 | 10.40 |
| AD | Aggression | Working memory | 3.00 | -0.26 | 0.11 | 37.94 | 0.05 | 1.47 | 0.195 | -0.96 | 0.45 |
| AD | Circadian rhythms | Attention | 2.00 | -2.35 | 2.03 | 97.54 | 8.05 | 1.00 | 0.454 | -28.10 | 23.50 |
| AD | Circadian rhythms | Global cognition | 5.00 | -0.43 | 0.36 | 89.92 | 0.19 | 3.79 | 0.299 | -1.46 | 0.59 |
| AD | Dysexecutive | Executive function | 5.00 | -0.07 | 0.16 | 57.01 | 0.07 | 3.43 | 0.683 | -0.53 | 0.39 |
| AD | Dysexecutive | Global cognition | 9.00 | -0.23 | 0.13 | 68.58 | 0.08 | 7.18 | 0.122 | -0.54 | 0.08 |
| AD | Dysexecutive | Working memory | 3.00 | -0.04 | 0.05 | 0.00 | 0.00 | 1.17 | 0.55 | -0.50 | 0.42 |
| AD | Motor disturbances | Attention | 2.00 | -2.50 | 2.35 | 97.64 | 10.74 | 1.00 | 0.48 | -32.30 | 27.30 |
| AD | Motor disturbances | Executive function | 4.00 | -0.19 | 0.07 | 21.56 | 0.02 | 1.84 | 0.133 | -0.54 | 0.16 |
| AD | Motor disturbances | Global cognition | 12.00 | -0.48 | 0.14 | 84.44 | 0.12 | 10.20 | 0.01 | -0.79 | -0.17 |
| AD | Motor disturbances | Working memory | 2.00 | -0.27 | 0.01 | 0.00 | 0.00 | 1 | 0.029 | -0.42 | -0.11 |
| AD | Overall NPS | Attention | 3.00 | -0.18 | 0.00 | 0.00 | 0.00 | 1.08 | 0.0137 | -0.23 | -0.13 |
| AD | Overall NPS | Executive function | 7.00 | -0.29 | 0.06 | 55.89 | 0.06 | 3.64 | 0.011 | -0.47 | -0.12 |
| AD | Overall NPS | Global cognition | 14.00 | -0.28 | 0.07 | 70.06 | 0.03 | 10.60 | 0.00209 | -0.44 | -0.13 |
| AD | Overall NPS | Memory | 3.00 | -0.21 | 0.03 | 2.08 | 0.00 | 1.74 | 0.0227 | -0.34 | -0.08 |
| AD | Overall NPS | Semantic knowledge | 3.00 | -0.15 | 0.03 | 0.00 | 0.00 | 1.79 | 0.0588 | -0.32 | 0.02 |
| AD | Overall NPS | Visuospatial skills | 2.00 | -0.14 | 0.00 | 0.00 | 0.00 | 1.00 | 0.00474 | -0.15 | -0.12 |
| AD | Overall NPS | Working memory | 2.00 | -0.30 | 0.08 | 0.00 | 0.00 | 1.00 | 0.169 | -1.32 | 0.73 |
| AD | Psychosis | Attention | 6.00 | -0.44 | 0.41 | 89.75 | 0.61 | 4.93 | 0.335 | -1.51 | 0.63 |
| AD | Psychosis | Executive function | 9.00 | -0.15 | 0.09 | 57.52 | 0.06 | 7.13 | 0.133 | -0.35 | 0.06 |
| AD | Psychosis | Global cognition | 27.00 | -0.42 | 0.08 | 85.03 | 0.10 | 24.50 | 0.000015 | -0.58 | -0.26 |
| AD | Psychosis | Memory | 8.00 | -0.14 | 0.08 | 57.42 | 0.06 | 6.25 | 0.114 | -0.33 | 0.05 |
| AD | Psychosis | Semantic knowledge | 9.00 | -0.18 | 0.09 | 48.30 | 0.04 | 6.72 | 0.0748 | -0.38 | 0.02 |
| AD | Psychosis | Speed | 2.00 | 0.12 | 0.15 | 0.00 | 0.00 | 1.00 | 0.589 | -1.84 | 2.07 |
| AD | Psychosis | Visuospatial skills | 7.00 | -0.02 | 0.07 | 0.00 | 0.00 | 4.76 | 0.839 | -0.20 | 0.17 |
| AD | Psychosis | Working memory | 5.00 | -0.29 | 0.06 | 0.00 | 0.00 | 1.83 | 0.0507 | -0.58 | 0.00 |
| AD | Aggression | Attention | 3.00 | -1.80 | 1.70 | 96.99 | 8.53 | 2.00 | 0.401 | -9.11 | 5.11 |
| AD | Aggression | Executive function | 4.00 | 0.04 | 0.04 | 0.00 | 0.00 | 1.24 | 0.51 | -0.29 | 0.36 |
| AD | Aggression | Global cognition | 9.00 | -0.43 | 0.23 | 82.91 | 0.18 | 7.33 | 0.103 | -0.97 | 0.11 |
| AD | Aggression | Memory | 2.00 | -0.47 | 0.54 | 77.81 | 0.57 | 1.00 | 0.541 | -7.26 | 6.32 |
| AD | Aggression | Semantic knowledge | 2.00 | -0.71 | 0.92 | 89.06 | 1.73 | 1.00 | 0.582 | -12.40 | 11.00 |
| AD | Aggression | Working memory | 3.00 | -0.14 | 0.14 | 37.99 | 0.05 | 1.46 | 0.455 | -1.00 | 0.73 |
| AD | Agitation | Executive function | 2.00 | -0.38 | 0.32 | 83.27 | 0.18 | 1.00 | 0.445 | -4.47 | 3.71 |
| AD | Agitation | Global cognition | 6.00 | -0.27 | 0.08 | 53.20 | 0.02 | 4.52 | 0.0214 | -0.48 | -0.06 |
| AD | Anxiety | Attention | 2.00 | -0.81 | 0.91 | 91.68 | 1.53 | 1.00 | 0.537 | -12.40 | 10.80 |
| AD | Anxiety | Executive function | 3.00 | -0.12 | 0.06 | 0.53 | 0.00 | 1.16 | 0.268 | -0.67 | 0.43 |
| AD | Anxiety | Global cognition | 8.00 | 0.03 | 0.06 | 0.00 | 0.00 | 3.90 | 0.664 | -0.14 | 0.20 |
| AD | Anxiety | Working memory | 2.00 | 0.20 | 0.36 | 77.60 | 0.21 | 1.00 | 0.678 | -4.41 | 4.81 |
| AD | Apathy | Attention | 4.00 | -0.10 | 0.10 | 0.00 | 0.00 | 2.80 | 0.377 | -0.42 | 0.22 |
| AD | Apathy | Executive function | 13.00 | -0.11 | 0.09 | 61.45 | 0.09 | 10.10 | 0.268 | -0.31 | 0.10 |
| AD | Apathy | Global cognition | 18.00 | -0.27 | 0.08 | 49.27 | 0.04 | 13.30 | 0.0461 | -0.44 | -0.10 |
| AD | Apathy | Memory | 8.00 | -0.15 | 0.04 | 0.00 | 0.00 | 2.40 | 0.0539 | -0.30 | 0.01 |
| AD | Apathy | Semantic knowledge | 8.00 | -0.20 | 0.07 | 0.00 | 0.00 | 2.25 | 0.0919 | -0.46 | 0.07 |
| AD | Apathy | Social cognition | 2.00 | 0.02 | 0.37 | 52.75 | 0.25 | 1.00 | 0.962 | -4.64 | 4.68 |
| AD | Apathy | Speed | 5.00 | -0.10 | 0.08 | 33.87 | 0.03 | 2.69 | 0.279 | -0.36 | 0.16 |
| AD | Apathy | Visuospatial skills | 4.00 | -0.15 | 0.08 | 0.00 | 0.00 | 1.87 | 0.214 | -0.54 | 0.23 |
| AD | Apathy | Working memory | 8.00 | -0.19 | 0.08 | 0.00 | 0.00 | 2.18 | 0.126 | -0.51 | 0.12 |
| AD | Delusions | Attention | 5.00 | -0.07 | 0.16 | 36.65 | 0.04 | 3.57 | 0.692 | -0.53 | 0.40 |
| AD | Delusions | Executive function | 7.00 | -0.25 | 0.11 | 51.13 | 0.04 | 5.04 | 0.08 | -0.54 | 0.04 |
| AD | Delusions | Global cognition | 18.00 | -0.29 | 0.07 | 69.27 | 0.05 | 15.20 | 0.00065 | -0.44 | -0.15 |
| AD | Delusions | Memory | 6.00 | -0.11 | 0.09 | 45.15 | 0.03 | 4.13 | 0.286 | -0.37 | 0.14 |
| AD | Delusions | Semantic knowledge | 7.00 | -0.18 | 0.08 | 44.01 | 0.03 | 4.88 | 0.0796 | -0.39 | 0.03 |
| AD | Delusions | Visuospatial skills | 5.00 | 0.03 | 0.06 | 0.00 | 0.00 | 3.22 | 0.678 | -0.15 | 0.20 |
| AD | Delusions | Working memory | 3.00 | -0.34 | 0.14 | 12.88 | 0.01 | 1.35 | 0.192 | -1.32 | 0.64 |
| AD | Depression | Attention | 6.00 | -0.23 | 0.07 | 7.12 | 0.00 | 1.68 | 0.1 | -0.59 | 0.13 |
| AD | Depression | Executive function | 9.00 | 0.17 | 0.29 | 91.77 | 0.30 | 7.82 | 0.58 | -0.50 | 0.83 |
| AD | Depression | Global cognition | 21.00 | 0.01 | 0.04 | 31.37 | 0.01 | 11.50 | 0.86 | -0.08 | 0.09 |
| AD | Depression | Memory | 8.00 | 0.26 | 0.19 | 83.88 | 0.13 | 6.54 | 0.22 | -0.20 | 0.72 |
| AD | Depression | Semantic knowledge | 5.00 | -0.05 | 0.12 | 67.13 | 0.03 | 3.08 | 0.714 | -0.42 | 0.33 |
| AD | Depression | Speed | 4.00 | -0.28 | 0.30 | 67.87 | 0.22 | 2.90 | 0.423 | -1.23 | 0.68 |
| AD | Depression | Visuospatial skills | 7.00 | 0.23 | 0.35 | 91.96 | 0.51 | 5.95 | 0.533 | -0.63 | 1.09 |
| AD | Depression | Working memory | 4.00 | 0.05 | 0.05 | 11.62 | 0.01 | 1.65 | 0.462 | -0.24 | 0.34 |
| AD | Disinhibition | Executive function | 4.00 | -0.05 | 0.18 | 61.00 | 0.08 | 2.65 | 0.808 | -0.68 | 0.58 |
| AD | Disinhibition | Global cognition | 8.00 | -0.23 | 0.15 | 72.84 | 0.09 | 6.45 | 0.156 | -0.58 | 0.12 |
| AD | Disinhibition | Working memory | 2.00 | 0.02 | 0.01 | 0.00 | 0.00 | 1.00 | 0.258 | -0.08 | 0.11 |
| AD | Dysphoria | Executive function | 2.00 | 0.10 | 0.31 | 45.45 | 0.09 | 1.00 | 0.804 | -3.81 | 4.01 |
| AD | Dysphoria | Global cognition | 3.00 | -0.12 | 0.10 | 0.00 | 0.00 | 1.35 | 0.402 | -0.86 | 0.61 |
| AD | Eating disturbances | Global cognition | 3.00 | -0.17 | 0.06 | 0.00 | 0.00 | 1.26 | 0.164 | -0.61 | 0.28 |
| AD | Euphoria | Executive function | 2.00 | 0.05 | 0.07 | 0.00 | 0.00 | 1.00 | 0.619 | -0.90 | 1.00 |
| AD | Euphoria | Global cognition | 5.00 | -0.02 | 0.05 | 0.00 | 0.00 | 2.46 | 0.715 | -0.22 | 0.18 |
| AD | Hallucinations | Attention | 3.00 | -1.29 | 1.31 | 94.72 | 2.30 | 1.99 | 0.429 | -6.93 | 4.35 |
| AD | Hallucinations | Executive function | 4.00 | -0.14 | 0.06 | 50.55 | 0.05 | 2.48 | 0.115 | -0.36 | 0.07 |
| AD | Hallucinations | Global cognition | 13.00 | -0.44 | 0.17 | 86.87 | 0.17 | 11.50 | 0.023 | -0.82 | -0.07 |
| AD | Hallucinations | Memory | 2.00 | -0.29 | 0.16 | 76.08 | 0.12 | 1.00 | 0.324 | -2.31 | 1.74 |
| AD | Hallucinations | Semantic knowledge | 2.00 | -0.25 | 0.23 | 70.70 | 0.08 | 1.00 | 0.484 | -3.23 | 2.73 |
| AD | Hallucinations | Working memory | 3.00 | -0.30 | 0.01 | 0.00 | 0.00 | 1.26 | 0.0144 | -0.41 | -0.19 |
| AD | Irritability | Executive function | 2.00 | 0.28 | 0.04 | 0.00 | 0.00 | 1.00 | 0.0902 | -0.22 | 0.78 |
| AD | Irritability | Global cognition | 4.00 | -0.02 | 0.10 | 0.00 | 0.00 | 1.88 | 0.841 | -0.48 | 0.44 |
| AD | Sleep disturbances | Global cognition | 2.00 | 0.03 | 0.02 | 0.00 | 0.00 | 1.00 | 0.296 | -0.17 | 0.24 |
| DLB | Affective | Executive function | 2.00 | -0.06 | 0.11 | 30.10 | 0.03 | 1.00 | 0.684 | -1.46 | 1.34 |
| DLB | Affective | Global cognition | 2.00 | -0.14 | 0.06 | 0.00 | 0.00 | 1.00 | 0.256 | -0.89 | 0.61 |
| DLB | Affective | Working memory | 2.00 | 0.10 | 0.04 | 37.63 | 0.04 | 1.00 | 0.264 | -0.45 | 0.64 |
| DLB | Overall NPS | Attention | 2.00 | -0.15 | 0.18 | 0.00 | 0.00 | 1.00 | 0.55 | -2.44 | 2.14 |
| DLB | Overall NPS | Executive function | 2.00 | -0.15 | 0.18 | 45.24 | 0.09 | 1.00 | 0.552 | -2.45 | 2.15 |
| DLB | Overall NPS | Global cognition | 2.00 | -0.19 | 0.18 | 0.00 | 0.00 | 1.00 | 0.488 | -2.52 | 2.14 |
| DLB | Overall NPS | Working memory | 2.00 | 0.14 | 0.12 | 0.00 | 0.00 | 1.00 | 0.468 | -1.42 | 1.70 |
| DLB | Psychosis | Global cognition | 3.00 | -0.47 | 0.19 | 36.20 | 0.08 | 1.88 | 0.145 | -1.35 | 0.42 |
| DLB | Apathy | Executive function | 2.00 | 0.07 | 0.05 | 0.00 | 0.00 | 1.00 | 0.361 | -0.53 | 0.67 |
| DLB | Apathy | Global cognition | 2.00 | -0.20 | 0.14 | 0.00 | 0.00 | 1.00 | 0.381 | -1.95 | 1.55 |
| DLB | Apathy | Working memory | 2.00 | -0.13 | 0.22 | 34.51 | 0.03 | 1.00 | 0.658 | -2.96 | 2.69 |
| DLB | Delusions | Global cognition | 2.00 | -0.72 | 0.08 | 0.00 | 0.00 | 1.00 | 0.0694 | -1.72 | 0.28 |
| DLB | Hallucinations | Global cognition | 2.00 | -0.30 | 0.17 | 0.00 | 0.00 | 1.00 | 0.333 | -2.50 | 1.90 |
| PD | Affective | Global cognition | 2.00 | -0.15 | 0.26 | 82.09 | 0.33 | 1.00 | 0.657 | -3.40 | 3.09 |
| PD | Psychosis | Attention | 2.00 | 0.14 | 0.14 | 87.22 | 0.94 | 1.00 | 0.516 | -1.67 | 1.95 |
| PD | Psychosis | Executive function | 2.00 | -0.02 | 0.26 | 71.20 | 0.35 | 1.00 | 0.95 | -3.37 | 3.33 |
| PD | Psychosis | Global cognition | 3.00 | -0.39 | 0.17 | 55.64 | 0.06 | 1.73 | 0.16 | -1.22 | 0.44 |
| PD | Psychosis | Memory | 2.00 | -0.24 | 0.53 | 81.76 | 0.65 | 1.00 | 0.726 | -6.94 | 6.46 |
| PD | Psychosis | Semantic knowledge | 2.00 | -0.21 | 0.61 | 82.98 | 0.65 | 1.00 | 0.79 | -7.97 | 7.55 |
| PD | Psychosis | Visuospatial skills | 2.00 | 0.11 | 0.03 | 0.00 | 0.00 | 1.00 | 0.172 | -0.29 | 0.52 |
| PD | Psychosis | Working memory | 2.00 | 0.01 | 0.08 | 0.00 | 0.00 | 1.00 | 0.903 | -0.98 | 1.00 |
| PD | Hallucinations | Global cognition | 3.00 | -0.39 | 0.17 | 55.64 | 0.06 | 1.73 | 0.16 | -1.22 | 0.44 |
