# Supplementary material for: The Associations Between Neuropsychiatric Symptoms and Cognition in People with Dementia: A Systematic Review and Meta-Analysis
Source: Neuropsychol Rev. 2023 Jul 21;34(2):581–97. doi: 10.1007/s11065-023-09608-0 (PMC11166771; doi:10.1007/s11065-023-09608-0)
Supplement: Supplementary file 5 — Supplementary file5 (DOCX 42 KB) [file 11065_2023_9608_MOESM5_ESM.docx]

**The associations between neuropsychiatric symptoms and cognition in people with dementia: A systematic review and meta-analysis**

*Neuropsychology review*

Ms. Julieta Sabates, The University of Melbourne, Australia.

Ms. Wei-Hsuan Chiu, The University of Melbourne, Australia.

A/Prof Samantha Loi, The University of Melbourne, Royal Melbourne Hospital, Australia.

Dr. Amit Lampit, The University of Melbourne, Australia.

Dr. Hanna M Gavelin, The University of Melbourne, Australia; Department of Psychology, Umea University, Sweden.

Dr. Terence Chong, The University of Melbourne, St Vincent’s Hospital Melbourne, Royal Melbourne Hospital, Australia.

Ms. Nathalie Launder, The University of Melbourne, Australia.

Dr.Anita MY Goh, National Ageing Research Institute; The University of Melbourne, Australia.

Prof. Amy Brodtmann, Cognitive Health Initiative, Central Clinical School, Monash University, Australia.

Prof. Nicola Lautenschlager, The University of Melbourne, Australia.

A/Prof. Alex Bahar-Fuchs, The University of Melbourne, Australia.

Corresponding author: Ms Julieta Sabates. Mailing address: 151 Barry Street, Carlton 3053, Victoria, Australia; Email address: Julieta.sabates@unimelb.edu.au

Supplementary material: S5 – Included studies references:

1. Akyol, M. A., Küçükgüçlü, Ö., & Yener, G. (2020). Investigation of factors affecting apathy in three major types of dementia. *Archives of Neuropsychiatry*, *57*(2), 120.
2. Balci, B. D., YENER, G., & Angin, S. (2011). The relationship between physical performance, cognition and depression in alzheimer type of dementia. *Journal of Neurological Sciences (Turkish)*, *28*(1), 051-057.
3. Benedict, R. H., Dobraski, M., & Goldstein, M. Z. (1999). A preliminary study of the association between changes in mood and cognition in a mixed geriatric psychiatry sample. *The Journals of Gerontology Series B: Psychological Sciences and Social Sciences*, *54*(2), P94-P99.
4. Bhat, A., Biswas, A., Das, G., Lahiri, D., Dubey, S., & Mukherjee, A. (2021). Behavioral variations among vascular cognitive impairment subtypes–A comparative study. *Applied Neuropsychology: Adult*, 1-8.
5. Breitve, M. H., Brønnick, K., Chwiszczuk, L. J., Hynninen, M. J., Aarsland, D., & Rongve, A. (2018). Apathy is associated with faster global cognitive decline and early nursing home admission in dementia with Lewy bodies. *Alzheimer's Research & Therapy*, *10*(1), 1-8.
6. Bronnick, K., Emre, M., Tekin, S., Haugen, S. B., & Aarsland, D. (2011). Cognitive correlates of visual hallucinations in dementia associated with Parkinson's disease. *Movement Disorders*, *26*(5), 824-829.
7. Bylsma, F. W., Richards, M., & Stern, Y. (1994). Delusions and patterns of cognitive impairment in Alzheimer’s disease.
8. Camargo, C. H. F., Serpa, R. A., Jobbins, V. A., Berbetz, F. A., & Sabatini, J. S. (2018). Differentiating between apathy and depression in patients with parkinson disease dementia. *American Journal of Alzheimer's Disease & Other Dementias®*, *33*(1), 30-34.
9. Chwiszczuk, L., Breitve, M. H., Brønnick, K., Gjerstad, M. D., Hynninen, M., Aarsland, D., & Rongve, A. (2017). REM sleep behavior disorder is not associated with a more rapid cognitive decline in mild dementia. *Frontiers in Neurology*, *8*, 375.
10. Contador-Castillo, I., Fernandez-Calvo, B., Cacho-Gutierrez, L. J., Ramos-Campos, F., & Hernandez-Martin, L. (2009). Depression in Alzheimer type-dementia: is there any effect on memory performance. *Revista de Neurología*, *49*(10), 505-510.
11. D'Antonio, F., Di Vita, A., Zazzaro, G., Brusà, E., Trebbastoni, A., Campanelli, A., ... & Boccia, M. (2019). Psychosis of Alzheimer's disease: Neuropsychological and neuroimaging longitudinal study. *International Journal of Geriatric Psychiatry*, *34*(11), 1689-1697.
12. de Oliveira, F. F., Machado, F. C., Sampaio, G., Marin, S. D. M. C., da Graça Naffah-Mazzacoratti, M., & Bertolucci, P. H. F. (2020). Neuropsychiatric feature profiles of patients with Lewy body dementia. *Clinical Neurology and Neurosurgery*, *194*, 105832.
13. de Oliveira, F. F., Wajman, J. R., Bertolucci, P. H. F., Chen, E. S., & Smith, M. C. (2015). Correlations among cognitive and behavioural assessments in patients with dementia due to Alzheimer's disease. *Clinical Neurology and Neurosurgery*, *135*, 27-33.
14. de Paula, J. J., Bicalho, M. A., Ávila, R. T., Cintra, M. T., Diniz, B. S., Romano-Silva, M. A., & Malloy-Diniz, L. F. (2016). A reanalysis of cognitive-functional performance in older adults: Investigating the interaction between normal aging, mild cognitive impairment, mild Alzheimer's disease dementia, and depression. *Frontiers in psychology*, *6*, 2061.
15. DeMichele-Sweet, M. A. A., Lopez, O. L., & Sweet, R. A. (2011). Psychosis in Alzheimer's disease in the national Alzheimer's disease coordinating center uniform data set: clinical correlates and association with apolipoprotein e. *International journal of Alzheimer’s disease*, *2011*.
16. Drijgers, R. L., Verhey, F. R., Leentjens, A. F., Köhler, S., & Aalten, P. (2011). Neuropsychological correlates of apathy in mild cognitive impairment and Alzheimer's disease: the role of executive functioning. *International Psychogeriatrics*, *23*(8), 1327-1333.
17. Eikelboom, W. S., van den Berg, E., Singleton, E. H., Baart, S. J., Coesmans, M., Leeuwis, A. E., ... & Papma, J. M. (2021). Neuropsychiatric and cognitive symptoms across the Alzheimer disease clinical spectrum: cross-sectional and longitudinal associations. *Neurology*, *97*(13), e1276-e1287.
18. Eustace, A., Kidd, N., Greene, E., Fallon, C., Bhrain, S. N., Cunningham, C., ... & Lawlor, B. A. (2001). Verbal aggression in Alzheimer's disease. Clinical, functional and neuropsychological correlates. *International Journal of Geriatric Psychiatry*, *16*(9), 858-861.
19. Fahlander, K., Berger, A. K., Wahlin, Å., & Bäckman, L. (1999). Depression does not aggravate the episodic memory deficits associated with Alzheimer's disease. *Neuropsychology*, *13*(4), 532.
20. Fernández, M., Gobartt, A. L., & Balañá, M. (2010). Behavioural symptoms in patients with Alzheimer's disease and their association with cognitive impairment. *BMC neurology*, *10*(1), 1-9.
21. Fernandez-Martinez, M., Molano, A., Castro, J., & J Zarranz, J. (2010). Prevalence of neuropsychiatric symptoms in mild cognitive impairment and Alzheimer's disease, and its relationship with cognitive impairment. *Current Alzheimer Research*, *7*(6), 517-526.
22. Fillit, H., Aigbogun, M. S., Gagnon‐Sanschagrin, P., Cloutier, M., Davidson, M., Serra, E., ... & Grossberg, G. (2021). Impact of agitation in long‐term care residents with dementia in the United States. *International Journal of Geriatric Psychiatry*, *36*(12), 1959-1969.
23. Fitz, A. G., & Teri, L. (1994). Depression, cognition, and functional ability in patients with Alzheimer's disease. *Journal of the American Geriatrics Society*, *42*(2), 186-191.
24. Flynn, F. G., Cummings, J. L., & Gornbein, J. (1991). Delusions in dementia syndromes: investigation of behavioral and neuropsychological correlates. *The Journal of neuropsychiatry and clinical neurosciences*.
25. Gallassi, R., Morreale, A., & Pagni, P. (2001). The relationship between depression and cognition. *Archives of Gerontology and Geriatrics*, *33*, 163-171.
26. Gallo, J. L., Schmidt, K. S., & Libon, D. J. (2008). Behavioral and psychological symptoms, neurocognitive performance, and functional independence in mild dementia. *Dementia*, *7*(3), 397-413.
27. Galynker, I. I., Roane, D. M., Miner, C. R., Feinberg, T. E., & Watts, P. (1995). Negative symptoms in patients with Alzheimer's disease. *The American Journal of Geriatric Psychiatry*, *3*(1), 52-59.
28. Gilley, D. W., Whalen, M. E., Wilson, R. S., & Bennett, D. A. (1991). Hallucinations and associated factors in Alzheimer's disease. *The Journal of neuropsychiatry and clinical neurosciences*.
29. Grossi, D., Santangelo, G., Barbarulo, A. M., Vitale, C., Castaldo, G., Proto, M. G., ... & Trojano, L. (2013). Apathy and related executive syndromes in dementia associated with Parkinson's disease and in Alzheimer's disease. *Behavioural neurology*, *27*(4), 515-522.
30. Hallikainen, I., Koivisto, A. M., Paajanen, T., Hiltunen, A., Karppi, P., Vanhanen, M., ... & Hänninen, T. (2012). Cognitive and neuropsychiatric symptom differences in early stages of Alzheimer’s disease: Kuopio ALSOVA study. *Dementia and Geriatric Cognitive Disorders Extra*, *2*(1), 209-218.
31. Harwood, D. G., Barker, W. W., Ownby, R. L., & Duara, R. (2000). Relationship of behavioral and psychological symptoms to cognitive impairment and functional status in Alzheimer's disease. *International Journal of Geriatric Psychiatry*, *15*(5), 393-400.
32. Hopkins, M. W., & Libon, D. J. (2005). Neuropsychological functioning of dementia patients with psychosis. *Archives of clinical neuropsychology*, *20*(6), 771-783.
33. Ito, T., Meguro, K., Akanuma, K., Meguro, M., Lee, E., Kasuya, M., ... & Mori, E. (2007). Behavioral and psychological symptoms assessed with the BEHAVE-AD-FW are differentially associated with cognitive dysfunction in Alzheimer’s disease. *Journal of Clinical Neuroscience*, *14*(9), 850-855.
34. Janzing, J. G., Naarding, P., & Eling, P. A. (2005). Depressive symptom quality and neuropsychological performance in dementia. *International Journal of Geriatric Psychiatry: A journal of the psychiatry of late life and allied sciences*, *20*(5), 479-484.
35. Keator, L. M., Wright, A. E., Saxena, S., Kim, K., Demsky, C., Sebastian, R., ... & Tippett, D. C. (2019). Distinguishing logopenic from semantic & nonfluent variant primary progressive aphasia: Patterns of linguistic and behavioral correlations. *Neurocase*, *25*(3-4), 98-105.
36. Kuzis, G., Sabe, L., Tiberti, C., Dorrego, F., & Starkstein, S. E. (1999). Neuropsychological correlates of apathy and depression in patients with dementia. *Neurology*, *52*(7), 1403-1403.
37. Kwak, Y. T., Yang, Y., Kwak, S. G., & Koo, M. S. (2013). Delusions of Korean patients with Alzheimer's disease: Study of drug‐naïve patients. *Geriatrics & Gerontology International*, *13*(2), 307-313.
38. Lam, C. L., Chan, W. C., Mok, C. C., Li, S. W., & Lam, L. C. (2006). Validation of the Chinese Challenging Behaviour Scale: clinical correlates of challenging behaviours in nursing home residents with dementia. *International Journal of Geriatric Psychiatry: A journal of the psychiatry of late life and allied sciences*, *21*(8), 792-799.
39. Lee, C. H., Kim, D. H., & Moon, Y. S. (2019). Differential associations between depression and cognitive function in MCI and AD: a cross-sectional study. *International psychogeriatrics*, *31*(8), 1151-1158.
40. Lee, C. H., Kim, D. H., & Moon, Y. S. (2019). Differential associations between depression and cognitive function in MCI and AD: a cross-sectional study. *International psychogeriatrics*, *31*(8), 1151-1158.
41. Lee, E., Meguro, K., Hashimoto, R., Meguro, M., Ishii, H., Yamaguchi, S., & Mori, E. (2007). Confabulations in episodic memory are associated with delusions in Alzheimer’s disease. *Journal of geriatric psychiatry and neurology*, *20*(1), 34-40.
42. Lee, W. J., Tsai, C. F., Gauthier, S., Wang, S. J., & Fuh, J. L. (2012). The association between cognitive impairment and neuropsychiatric symptoms in patients with Parkinson's disease dementia. *International psychogeriatrics*, *24*(12), 1980-1987.
43. Logsdon, R. G., Teri, L., McCurry, S. M., Gibbons, L. E., Kukull, W. A., & Larson, E. B. (1998). Wandering: a significant problem among community residing individuals with Alzheimer's disease. *The Journals of Gerontology Series B: Psychological Sciences and Social Sciences*, *53*(5), P294-P299.
44. Lopez, O. L., Becker, J. T., Brenner, R. P., Rosen, J., Bajulaiye, O. I., & Reynolds, C. F. (1991). Alzheimer's disease with delusions and hallucinations: neuropsychological and electroencephalographic correlates. *Neurology*, *41*(6), 906-906.
45. Machado, F. C., Oliveira, F. F. D., Marin, S. D. M. C., Sampaio, G., & Bertolucci, P. H. F. (2020). Correlates of neuropsychiatric and motor tests with language assessment in patients with Lewy body dementia. *Archives of Clinical Psychiatry (São Paulo)*, *47*, 75-81.
46. Mariano, L. I., Caramelli, P., Guimarães, H. C., Gambogi, L. B., Moura, M. V. B., Yassuda, M. S., ... & de Souza, L. C. (2020). Can social cognition measurements differentiate behavioral variant frontotemporal dementia from Alzheimer’s disease regardless of apathy?. *Journal of Alzheimer's Disease*, *74*(3), 817-827.
47. McPherson, SFairbanks, L., Tiken, S., Cummings, J. L., & Back-Madruga, C. (2002). Apathy and executive function in Alzheimer's disease. *Journal of the International Neuropsychological Society*, *8*(3), 373-381.
48. Migliorelli, R., Petracca, G., Teson, A., Sabe, L., Leiguarda, R., & Starkstein, S. E. (1995). Neuropsychiatric and neuropsychological correlates of delusions in Alzheimer's disease. *Psychological medicine*, *25*(3), 505-513.
49. Mizrahi, R., Starkstein, S. E., Jorge, R., & Robinson, R. G. (2006). Phenomenology and clinical correlates of delusions in Alzheimer disease. *The American journal of geriatric psychiatry*, *14*(7), 573-581.
50. Montagnese, M., Vignando, M., Collerton, D., Ffytche, D., Mosimann, U. P., Taylor, J. P., ... & Urwyler, P. (2022). Cognition, hallucination severity and hallucination-specific insight in neurodegenerative disorders and eye disease. *Cognitive neuropsychiatry*, *27*(2-3), 105-121.
51. Na, H. R., Kang, D. W., Woo, Y. S., Bahk, W. M., Lee, C. U., & Lim, H. K. (2018). Relationship between Delusion of Theft and Cognitive Functions in Patients with Mild Alzheimer’s Disease. *Psychiatry Investigation*, *15*(4), 413.
52. Naarding, P., de Koning, I., van Kooten, F., Janzing, J. G., Beekman, A. T., & Koudstaal, P. J. (2007). Post‐stroke dementia and depression: frontosubcortical dysfunction as missing link?. *International Journal of Geriatric Psychiatry: A journal of the psychiatry of late life and allied sciences*, *22*(1), 1-8.
53. Nagata, T., Nakajima, S., Shinagawa, S., Plitman, E., Graff‐Guerrero, A., Mimura, M., & Nakayama, K. (2017). Psychosocial or clinico‐demographic factors related to neuropsychiatric symptoms in patients with Alzheimer's disease needing interventional treatment: analysis of the CATIE‐AD study. *International journal of geriatric psychiatry*, *32*(12), 1264-1271.
54. Nagata, T., Shinagawa, S., Ochiai, Y., Kada, H., Kasahara, H., Nukariya, K., & Nakayama, K. (2010). Relationship of frontal lobe dysfunction and aberrant motor behaviors in patients with Alzheimer's disease. *International psychogeriatrics*, *22*(3), 463-469.
55. Nakaaki, S., Murata, Y., Sato, J., Shinagawa, Y., Hongo, J., Tatsumi, H., ... & Furukawa, T. A. (2008). Association between apathy/depression and executive function in patients with Alzheimer's disease. *International Psychogeriatrics*, *20*(5), 964-975.
56. Nakaaki, S., Murata, Y., Sato, J., Shinagawa, Y., Tatsumi, H., Hirono, N., & Furukawa, T. A. (2007). Greater impairment of ability in the divided attention task is seen in Alzheimer’s disease patients with depression than in those without depression. *Dementia and Geriatric Cognitive Disorders*, *23*(4), 231-240.
57. Nakatsuka, M., Meguro, K., Nakamura, K., Akanuma, K., & Yamaguchi, S. (2014). ‘Residence Is Not Home'Is a Particular Type of Delusion Associated with Cognitive Decline of Alzheimer's Disease. *Dementia and geriatric cognitive disorders*, *38*(1-2), 46-54.
58. Oliveira, F. F., Machado, F. C., Sampaio, G., Marin, S. M., Chen, E. S., Smith, M. C., & Bertolucci, P. H. (2015). Contrasts between patients with Lewy body dementia syndromes and APOE-ε3/ε3 patients with late-onset Alzheimer disease dementia. *The neurologist*, *20*(2), 35-41.
59. Onyike, C. U., Sheppard, J. M. E., Tschanz, J. T., Norton, M. C., Green, R. C., Steinberg, M., ... & Lyketsos, C. G. (2007). Epidemiology of apathy in older adults: the Cache County Study. *The American journal of geriatric psychiatry*, *15*(5), 365-375.
60. Pagonabarraga, J., Llebaria, G., García‐Sánchez, C., Pascual‐Sedano, B., Gironell, A., & Kulisevsky, J. (2008). A prospective study of delusional misidentification syndromes in Parkinson's disease with dementia. *Movement disorders: official journal of the Movement Disorder Society*, *23*(3), 443-448.
61. Park, S., Kim, D. K., Myung, W., Yoo, J. H., Shin, S. J., Na, D. L., ... & Shin, J. (2019). Risk factors of behavioral and psychological symptoms in patients with Alzheimer disease: The clinical research of dementia of South Korea study. *Korean Journal of Family Medicine*, *40*(1), 16.
62. Perneczky, R., Drzezga, A., Boecker, H., Wagenpfeil, S., Förstl, H., Kurz, A., & Häussermann, P. (2009). Right prefrontal hypometabolism predicts delusions in dementia with Lewy bodies. *Neurobiology of aging*, *30*(9), 1420-1429.
63. Perri, R., Monaco, M., Fadda, L., Caltagirone, C., & Carlesimo, G. A. (2014). Neuropsychological correlates of behavioral symptoms in Alzheimer's disease, frontal variant of frontotemporal, subcortical vascular, and lewy body dementias: a comparative study. *Journal of Alzheimer's Disease*, *39*(3), 669-677.
64. Perri, R., Turchetta, C. S., Caruso, G., Fadda, L., Caltagirone, C., & Carlesimo, G. A. (2018). Neuropsychological correlates of cognitive, emotional-affective and auto-activation apathy in Alzheimer's disease. *Neuropsychologia*, *118*, 12-21.
65. Pezzoli, S., Cagnin, A., Antonini, A., & Venneri, A. (2019). Frontal and subcortical contribution to visual hallucinations in dementia with Lewy bodies and Parkinson’s disease. *Postgraduate Medicine*, *131*(7), 509-522.
66. Qian, W., Fischer, C. E., Schweizer, T. A., & Munoz, D. G. (2018). Association between psychosis phenotype and APOE genotype on the clinical profiles of Alzheimer's disease. *Current Alzheimer Research*, *15*(2), 187-194.
67. Quaranta, D., Vita, M. G., Bizzarro, A., Masullo, C., Piccininni, C., Gainotti, G., & Marra, C. (2015). Cognitive and behavioral determinants of psychotic symptoms in Alzheimer's disease. *Dementia and geriatric cognitive disorders*, *39*(3-4), 194-206.
68. Reed, B. R., Jagust, W. J., & Coulter, L. (1993). Anosognosia in Alzheimer's disease: relationships to depression, cognitive function, and cerebral perfusion. *Journal of clinical and experimental neuropsychology*, *15*(2), 231-244.
69. Rochat, L., Billieux, J., Van der Linden, A. C. J., Annoni, J. M., Zekry, D., Gold, G., & Van der Linden, M. (2013). A multidimensional approach to impulsivity changes in mild Alzheimer’s disease and control participants: Cognitive correlates. *Cortex*, *49*(1), 90-100.
70. Rolland, Y., Andrieu, S., Cantet, C., Morley, J. E., Thomas, D., Nourhashemi, F., & Vellas, B. (2007). Wandering behavior and Alzheimer disease. The REAL. FR prospective study. *Alzheimer Disease & Associated Disorders*, *21*(1), 31-38.
71. Ross, L. K., Arnsberger, P., & Fox, P. J. (1998). The relationship between cognitive functioning and disease severity with depression in dementia of the Alzheimer's type. *Aging & Mental Health*, *2*(4), 319-327.
72. Rozum, W. J., Cooley, B., Vernon, E., Matyi, J., & Tschanz, J. T. (2019). Neuropsychiatric symptoms in severe dementia: Associations with specific cognitive domains the Cache County Dementia Progression Study. *International journal of geriatric psychiatry*, *34*(7), 1087-1094.
73. Ruiz, M., Arias, A., Sánchez-Llanos, E., Gil, M. P., López-Ortega, R., Dakterzada, F., ... & Piñol-Ripoll, G. (2018). Minor hallucinations in Alzheimer’s disease. *Journal of Alzheimer's Disease*, *64*(2), 543-549.
74. Sánchez-Rodríguez, JL. (2004). Neuropsychologic performance and depressed mood in sporadic late onset Alzheimer disease. *Revista de Neurologia*, *38*(7), 625-630.
75. Senanarong, V., Poungvarin, N., Jamjumras, P., Sriboonroung, A., Danchaivijit, C., Udomphanthuruk, S., & Cummings, J. L. (2005). Neuropsychiatric symptoms, functional impairment and executive ability in Thai patients with Alzheimer's disease. *International psychogeriatrics*, *17*(1), 81-90.
76. Serra, L., Perri, R., Fadda, L., Padovani, A., Lorusso, S., Pettenati, C., ... & Carlesimo, G. A. (2010). Relationship between cognitive impairment and behavioural disturbances in Alzheimer's disease patients. *Behavioural neurology*, *23*(3), 123-130.
77. Shin, H. Y., Han, H. J., Shin, D. J., Park, H. M., Lee, Y. B., & Park, K. H. (2014). Sleep problems associated with behavioral and psychological symptoms as well as cognitive functions in Alzheimer's disease. *Journal of Clinical Neurology*, *10*(3), 203-209.
78. Soleman Hernandez, S. S., Vital, T. M., Garuffi, M., Stein, A. M., Teixeira, C. V. L., Costa, J. L. R., & Stella, F. (2012). Apathy, cognitive function and motor function in Alzheimer's disease. *Dementia & Neuropsychologia*, *6*, 236-243.
79. Starkstein, S. E., Ingram, L., Garau, M. L., & Mizrahi, R. (2005). On the overlap between apathy and depression in dementia. *Journal of Neurology, Neurosurgery & Psychiatry*, *76*(8), 1070-1074.
80. Starr, J. M., & Lonie, J. (2007). Relationship between behavioural and psychological symptoms of dementia and cognition in Alzheimer’s disease. *Dementia and geriatric cognitive disorders*, *24*(5), 343-347.
81. Strauss, M. E., & Sperry, S. D. (2002). An informant-based assessment of apathy in Alzheimer disease. *Cognitive and Behavioral Neurology*, *15*(3), 176-183.
82. Sultzer, D. L., Leskin, L. P., Melrose, R. J., Harwood, D. G., Narvaez, T. A., Ando, T. K., & Mandelkern, M. A. (2014). Neurobiology of delusions, memory, and insight in Alzheimer disease. *The American Journal of Geriatric Psychiatry*, *22*(11), 1346-1355.
83. Sultzer, D. L., Levin, H. S., Mahler, M. E., High, W. M., & Cummings, J. L. (1992). Assessment of cognitive, psychiatric, and behavioral disturbances in patients with dementia: the Neurobehavioral Rating Scale. *Journal of the American Geriatrics Society*, *40*(6), 549-555.
84. Van der Mussele, S., Bekelaar, K., Le Bastard, N., Vermeiren, Y., Saerens, J., Somers, N., ... & Engelborghs, S. (2013). Prevalence and associated behavioral symptoms of depression in mild cognitive impairment and dementia due to Alzheimer's disease. *International Journal of Geriatric Psychiatry*, *28*(9), 947-958.
85. Van der Mussele, S., Le Bastard, N., Saerens, J., Somers, N., Mariën, P., Goeman, J., ... & Engelborghs, S. (2015). Agitation-associated behavioral symptoms in mild cognitive impairment and Alzheimer's dementia. *Aging & mental health*, *19*(3), 247-257.
86. Wagner, A. W., Teri, L., & Orr-Rainey, N. (1995). Behavior problems of residents with dementia in special care units. *Alzheimer disease and associated disorders*.
87. Welsh, S. W., Corrigan, F. M., & Scott, M. (1996). Language impairment and aggression in Alzheimer's disease. *International journal of geriatric psychiatry*, *11*(3), 257-261.
88. Wu, H. S. (2014). Predictors of hyperphagia in institutionalized patients with dementia. *Journal of Nursing Research*, *22*(4), 250-258.
89. Yeager, C. A., & Hyer, L. E. E. (2008). Apathy in dementia: relations with depression, functional competence, and quality of life. *Psychological reports*, *102*(3), 718-722.
90. Zahodne, L. B., Ornstein, K., Cosentino, S., Devanand, D. P., & Stern, Y. (2015). Longitudinal relationships between Alzheimer disease progression and psychosis, depressed mood, and agitation/aggression. *The American Journal of Geriatric Psychiatry*, *23*(2), 130-140.
